# Facile Lithium Densification Kinetics by Hyperporous/Hybrid Conductor for High‐Energy‐Density Lithium Metal Batteries

**DOI:** 10.1002/advs.202402156

**Published:** 2024-04-22

**Authors:** Dong‐Yeob Han, Saehun Kim, Seoha Nam, Gayoung Lee, Hongyeul Bae, Jin Hong Kim, Nam‐Soon Choi, Gyujin Song, Soojin Park

**Affiliations:** ^1^ Department of Chemistry Pohang University of Science and Technology (POSTECH) 77 Cheongam‐Ro, Nam‐gu Pohang Gyeongbuk 37673 Republic of Korea; ^2^ Department of Chemical and Biomolecular Engineering Korea Advanced Institute of Science and Technology (KAIST) 291 Daehak‐ro Yuseong‐gu Daejeon 34141 Republic of Korea; ^3^ Graduate Institute of Ferrous & Eco Materials Technology Pohang University of Science and Technology (POSTECH) 77 Cheongam‐Ro, Nam‐gu Pohang Gyeongbuk 37673 Republic of Korea; ^4^ Battery Materials R&D Laboratory POSCO Holdings, 67 Cheongam‐ro, Nam‐gu Pohang 37673 Republic of Korea; ^5^ Ulsan Advanced Energy Technology R&D Center Korea Institute of Energy Research (KIER) Ulsan 44776 Republic of Korea

**Keywords:** fast‐charging, lithium metal densification, lithium‐filling host, mixed conductor, nonsolvent‐induced phase separation

## Abstract

Lithium metal anode (LMA) emerges as a promising candidate for lithium (Li)‐based battery chemistries with high‐energy‐density. However, inhomogeneous charge distribution from the unbalanced ion/electron transport causes dendritic Li deposition, leading to “dead Li” and parasitic reactions, particularly at high Li utilization ratios (low negative/positive ratios in full cells). Herein, an innovative LMA structural model deploying a hyperporous/hybrid conductive architecture is proposed on single‐walled carbon nanotube film (HCA/C), fabricated through a nonsolvent induced phase separation process. This design integrates ionic polymers with conductive carbon, offering a substantial improvement over traditional metal current collectors by reducing the weight of LMA and enabling high‐energy‐density batteries. The HCA/C promotes uniform lithium deposition even under rapid charging (up to 5 mA cm^−2^) owing to its efficient mixed ion/electron conduction pathways. Thus, the HCA/C demonstrates stable cycling for 200 cycles with a low negative/positive ratio of 1.0 when paired with a LiNi_0.8_Co_0.1_Mn_0.1_O_2_ cathode (areal capacity of 5.0 mAh cm^−2^). Furthermore, a stacked pouch‐type full cell using HCA/C realizes a high energy density of 344 Wh kg^−1^_cell_/951 Wh L^−1^_cell_ based on the total mass of the cell, exceeding previously reported pouch‐type full cells. This work paves the way for LMA development in high‐energy‐density Li metal batteries.

## Graphical abstract

Lithium metal anode should satisfy significant requirements related to fast‐charging feasibility and high‐energy‐density battery design. The hyperporous/hybrid conductive electrode induces simultaneous lithium deposition and facile lithium densification kinetics at fast current density. Besides, carbon‐based ultra‐light architecture realizes dramatic weight reduction of lithium metal anode, compared with a typical metal current collector to effectively achieve high gravimetric energy density.

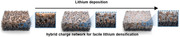

## 1 Introduction

High‐energy‐density batteries are essential to realize global electrification.^[^ ^1^ ^]^ Strategic approaches of an electroactive material design have been applied to raise up inherent electrochemical properties of lithium‐ion batteries (LIBs).^[^ ^2^ ^]^ Despite of remarkable progress in material development, there are some engineering issues in LIBs related to constructing a rational electrode such as a restriction of high‐loaded electrode fabrication and poor charge integrity between individual ingredients consisting of the electrode.^[^ ^3^ ^]^ Systematic approaches, such as a dry process for thick electrodes and tandem pressing process for enhanced integrity and reduced porosity, partially address electrode structure issues. However, these solutions ultimately encounter design limitations due to the trade‐off between material loading and energy density.^[^ ^4^ ^]^ Thus, building rational/efficient battery configuration is under challenge if maintaining the use of typical electroactive materials (e.g., graphite, silicon) and these bottlenecks have blocked the revolution of LIBs to achieve high‐energy‐density systems.

In this regard, the lithium metal anodes (LMAs) have been revisited as alternative electrodes, reducing variables to achieve the high‐energy‐density battery in the aspect of the production process owing to disparate electrode configuration.^[^ ^5^ ^]^ Lithium (Li) metal participates in electrochemical reactions through direct dissolution/deposition into/from an electrolyte as the electrode itself, compared to intercalation‐type (graphite) or alloy‐type (silicon) electroactive materials involving in ion diffusion term.^[^ ^6^ ^]^ Therefore, LMA makes a difference due to the as‐mentioned electrochemical distinction with other anode configurations when increasing gravimetric/volumetric energy density along with simply reducing the thickness or amounts of Li metal. Besides, intrinsic advantages exhibiting higher theoretical capacity (3860 mAh g^−1^) and lower reduction potential (−3.04 V versus standard hydrogen electrode, SHE) render LMA a more attractive anode rather than the as‐mentioned anode materials.^[^ ^7^ ^]^ For this reason, the LMA shows the feasibility as a suitable candidate for high‐energy‐density realization.

With those strong points, LMA has further adopted a thinner Li metal anode and a low negative/positive (N/P) ratio system to extremely increase the energy density.^[^ ^8^ ^]^ However, the Li metal shows inevitable electrochemical behavior that non‐uniform electrode/electrolyte interphase induces Li dendrite growth and infinite volume change, leading to battery failure.^[^ ^9^ ^]^ At this point, the challenging system with a high Li utilization ratio (ultrathin LMA or low N/P ratio) electrochemically accelerates the electrode degradation on cycling by early reach to a Li depletion stage. Moreover, film‐type Li metal operates an electrochemical reaction at only a two‐dimensional surface (low electrode/electrolyte contact volume). In other words, the general properties of LMA theoretically enable to build high energy density, however, persisting designed energy density and retarding the Li metal degradation on fast cycling are the most challenging issues to realize high‐energy‐density Li metal batteries (LMBs).^[^ ^10^ ^]^

Herein, we design a rational LMA model applying a technical architecture to ultimately demonstrate high‐energy‐density LMBs with stable and fast‐charge cycling achievements. This consists of anorganic‐based ionic polymer (polyvinyl alcohol, PVA)/conductive carbon (carbon black (CB) and single‐walled carbon nanotube (SWCNT)) complex that acts as both Li host and/or current collector by lithiophilic and electrical interconnection in a whole body.^[^ ^11^ ^]^ In this regard, it is significantly worth that the compositive network can substitute copper (Cu) or stainless‐steel foil and thus substantially reduce the weight of LMA toward a practical high‐energy‐density battery system as well as stable electrodeposition/dissolution operation.^[^ ^12^ ^]^ In addition, for further improvement in the aspect of the fast‐charging system, we cast the polymer/carbon composite dispersion to construct a hyperporous/hybrid conductive architecture (HCA) on SWCNT film (HCA/C) through nonsolvent induced phase separation (NIPS) technique by tuning hierarchical pores with nonsolvent optimization.^[^ ^13^ ^]^ This HCA/C efficiently accepts a high current flux for fast electrochemical kinetics, concretely that numerous Li‐absorbing/electron‐attracting sites in HCA/C allow for spontaneous electrodeposition with low nucleation and growth overpotential compared to Li foil or metal current collectors regardless of the current density. Li metal deposited in HCA/C (HCA/C‐Li) exhibits uniform and dense morphological structure under both low and high current densities – unlike Cu current collectors. It maintains stable electrochemical reactions under high‐energy and fast‐charging conditions, without notable structure degradation and fast Li depletion by consecutive Li and electrolyte consumption under a high utilization ratio of Li. Finally, we demonstrate that HCA‐Li shows high compatibility with various cathode conditions (maximum capacity: 5.0 mAh cm^−2^) and N/P ratio (minimum ratio: 1.0) in both coin‐type and stacked pouch‐type full cells under not plenty of electrolyte system (3.0 g Ah^−1^) for practical evaluation. Importantly, the resultant stacked pouch full cell achieved a high‐energy‐density (344 Wh kg_cell_^−1^/951 WhL_cell_^−1^, including all cell components), surpassing previously reported pouch‐type full cells. This advanced architecture enables efficient modulation of charge carriers into abundant active sites and retards the early‐stage depletion to extend the cycle life in high Li utilization ratio and high‐energy density battery systems.

## 2 Results and Discussion

### 2.1 Building Hyperporous and Hybrid Conductive Architecture

**Scheme 1:**
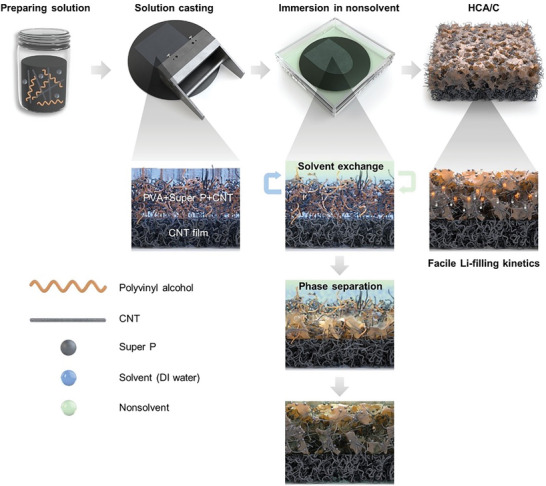
Fabrication process of hyperporous/hybrid conductive architecture on CNT film (HCA/C).

The fabrication process of HCA/C through the NIPS technique is illustrated in **Scheme** **1**. Dense and free‐standing SWCNT film was prepared as an electrical‐conductive substrate via vacuum filtration of well‐dispersive SWCNT aqueous solution to develop facile HCA on the top layer (Figure S1, Supporting Information). Meantime, CB/SWCNT dispersion as conductive agents was mixed with PVA aqueous solution to design a hybrid conductive system of HCA acting as both electronic/ionic charge passage in the whole structure. After making a water‐based slurry of CB/SWCNT/PVA complex, the slurry was cast on as‐prepared free‐standing SWCNT film using a doctor blade and subsequently experienced NIPS process through immersing it in nonsolvent. The solvent in the solution and nonsolvent are immediately exchanged, leading to the formation of polymer‐rich and polymer‐poor phases. These phases naturally segregate to form the structural framework and the porous matrix of the architecture, respectively. Subsequent vacuum drying at 70 °C for 6 hr ensures the complete removal of residual solvents and nonsolvents. The resultant porous structure of HCA/C facilitates electrolyte penetration and fast‐charging with enhanced Li‐filling kinetics.

**Figure 1:**
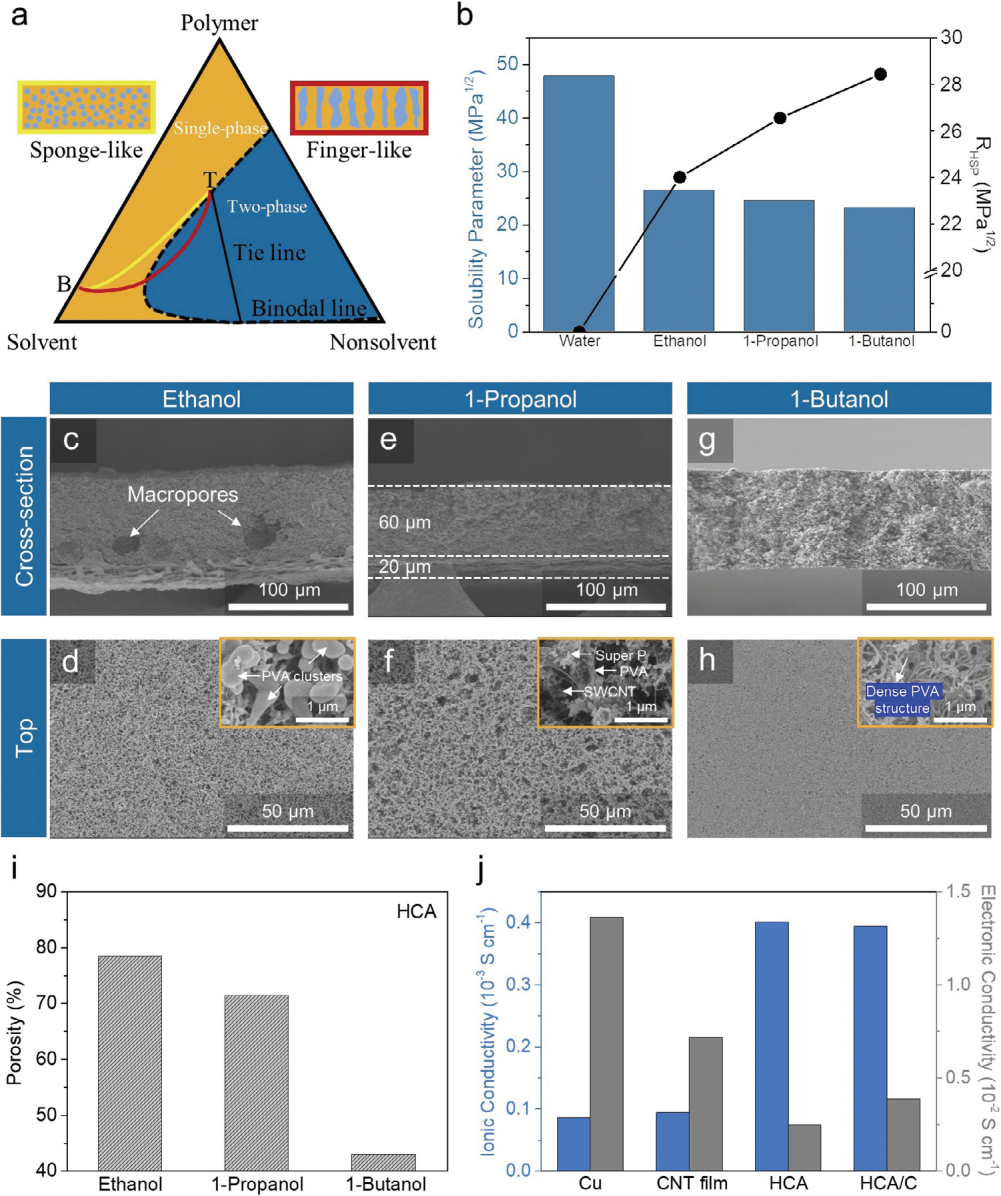
Structure‐forming design factors of HCA. a) Composition paths of a complex after immersion (t < 1s): yellow and red lines induce sponge‐like and finger‐like structures, respectively. b) Solubility parameters of solvent, nonsolvent, and *R*_HPS_ between solvent and nonsolvent. c, e, g) Cross‐sectional and d, f, h) top‐view SEM images of HCA/C fabricated by ethanol, 1‐propanol, and 1‐butanol nonsolvent (left‐to‐right) The insets of top‐view SEM images indicate magnified ones to confirm the formation structure of three components in architectures. i) Changes in porosity of HCA/C with different nonsolvent. j) Comparisons of electronic conductivity and electrolyte‐impregnated ionic conductivity for various electrodes.

The formation of pore structures via the NIPS technique is predominantly governed by the thermodynamic and kinetics of polymer chain interactions within the nonsolvent medium, modulated by three main components (i.e., polymer, solvent, and nonsolvent).^[^ ^14^ ^]^ Within the ternary phase diagram, which depicts the equilibrium between these components, the system is characterized by one‐phase and two‐phase regions separated by the binodal line, derived from Flory‐Huggins thermodynamic principles (**Figure** **1**a).^[^ ^14b^ ^]^ Upon the immersion of the as‐cast complex into the nonsolvent, a chemical potential gradient triggers a solvent exchange process between the solvent and nonsolvent within the coagulation bath. This exchange rate is pivotal in dictating the demixing behavior, subsequently resulting in varied pore architectures. An accelerated exchange rate drives the system from a one‐phase to a two‐phase region after immersion (red line), leading to a finger‐like structure with macropores. Conversely, a gradual exchange rate maintains the system within the initial phase (yellow line), yielding a sponge‐like morphology with micropores. The dynamics of this exchange are significantly influenced by the solvent‐nonsolvent interaction, highlighting the importance of selecting appropriate solvent‐nonsolvent pair to fabricate the desired porous structure. The thermodynamic compatibility between the solvent and nonsolvent is quantified using the Hansen solubility parameter (HSP), which encompasses three components: dispersion (δ_d_), polar (δ_p_), and hydrogen bonding (δ_h_) forces. The compatibility, or relative affinity, between solvent and nonsolvent is predicted by calculating the HSP distance (*R*_HSP_), using the following Equation (1).^[^ ^15^ ^]^

(1) $$\;R_{\mathrm{HSP}}=\;\sqrt{4{\left(\delta_{dS}-\delta_{dNS}\right)}^{2}+{\left((\delta_{pS}-\delta_{pNS}\right)}^{2}+{\left(\delta_{hS}-\delta_{hNS}\right)}^{2}}$$

where “S” and “NS” denote solvent and nonsolvent, respectively. In our system, various nonsolvents (ethanol, 1‐propanol, and 1‐butanol) were explored to optimize and design fine HCA structure from water‐based carbon black/SWCNT/PVA complex slurry, finally realizing proper pore creation toward both achievements of conductive network maintenance and facile Li metal‐filling kinetics. The calculated *R*_HSP_ values for water‐ethanol, water‐1‐propanol, and water‐1‐butanol systems were 24.0, 26.6, and 28.4, respectively, indicating varying degrees of miscibility and, by extension, different solvent exchange rates in the coagulation bath leading to distinct pore structures (Figure 1b, Table S1, Supporting Information).

Consequently, the HCA fabricated using ethanol as the nonsolvent exhibited macro‐size complex‐poor phase formation during the solvent exchange, resulting in micro‐level macropores and low densified structure (Figure 1c).^[^ ^15^, ^16^ ^]^ Besides, fast nonsolvent penetration resulted in aggregated PVA clusters because insufficient time was allowed to fabricate a rational porous scaffold (Figure 1d). Meanwhile, the 1‐propanol nonsolvent could relatively reduce the exchange rate owing to the bigger gap of solubility parameter with water, compared to ethanol nonsolvent. Thus, the structure‐forming reaction time was apparently guaranteed that the complex produced proper macro/mesopores in the whole structure without polymer aggregation (Figures 1e, and f). However, we observed that using 1‐butanol as a nonsolvent interrupted pore formation due to the immiscibility of butanol and water. Consequently, instead of the desired porous structure, we observed a film‐like, dense PVA structure (Figures 1g and h).

In considering the pore formation based on different nonsolvents, HCAs formed by three distinct nonsolvents exhibit a trend where more immiscible nonsolvents result in porosity decrease and smaller pore formation due to exchange kinetics (Figure 1i; Figure S2, Supporting Information). To compare the electrolyte‐impregnated ionic conductivity and electronic conductivity of various HCA/Cs with different nonsolvents, we conducted calculations using the results, derived from Nyquist plots and direct current‐voltage measurements (Figure S3, Equations 2 and 3, see details in the Experimental Section, Supporting Information).^[^ ^17^ ^]^ The HCA/C fabricated with ethanol as the nonsolvent displayed the lowest electronic conductivity, attributed to the presence of aggregated PVA clusters on the surface and poor carbon network by dominant macropores in the architecture. Conversely, the HCA/C fabricated with 1‐butanol, featuring the most dense structure, exhibited the highest electronic conductivity due to more closed packing of conductive components.

Regarding electrolyte‐impregnated ionic conductivities, they followed the order of the porosity of the structure, directly influencing the electrolyte‐impregnated ability. Compared with other current collectors(Cu foil and SWCNT film), both HCA and HCA/C showed higher electrolyte‐impregnated ionic conductivity owing to a larger contact area and ionic polymer presence in the structure (Figure 1j; Figure S4, Supporting Information). Besides, with the support of well‐distributed conductive agents in the complex, HCA/C exhibited competitive electronic conductivity to attract charge carriers in the whole porous structure upon fast electrochemical reaction (Figure 1h; Figure S5, Supporting Information). Furthermore, good interface linkage with the SWCNT bottom layer permitted the enhancement of electronic conductivity in the whole HCA/C structure (Figure S6, Supporting Information).

### 2.2 Fast‐Charging Feasibility of HCA/C Electrode

**Figure 2:**
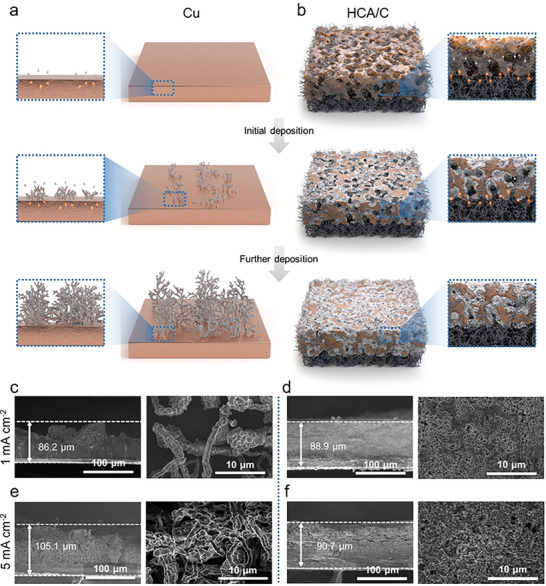
Fast‐charging feasibility of HCA/C. Schematic illustration of structural growth of lithium metal during electrochemical deposition on a) Cu foil and b) HCA/C. SEM images after lithium deposition of 5 mAh cm^−2^ on Cu foil (left) and in HCA/C (right) under current densities of c, d) 1 mA cm^−2^ and e, f) 5 mA cm^−2^.

The rational structure of HCA/C shows several advantages in fast Li densification kinetics and stable Li metal electrodeposition even under rapid current densities. The designated architecture simultaneously enables to facilitate the charge transfer in the whole structure which involves numerous electroactive sites and hyperporous network as well as realizes lithiophilic properties along with ionic and Li‐attractive PVA polymer by hydroxyl groups.^[^ ^18^ ^]^ Typically, the high difference of surface energy between Cu electrode and Li‐ions for Li metal nucleation at initial induced large overpotential (103 mV) hardly to create Li metal seeds on Cu and subsequent Li deposition was conducted around the Li seeds (Figure S7a, Supporting Information).^[^ ^19^ ^]^ Conversely, HCA/C exhibited electrochemical reactions early in charging, showing Li‐ion accommodation and a smooth surface for Li metal deposition with a mere 24 mV overpotential for nucleation and further Li metal deposition to 5 mAh cm^−2^ in this system.^[^ ^20^ ^]^ For this reason, there were no attractive forces on the Cu electrode that spread or densified Li metal leading to the form of dendritic‐grown Li metal anode (**Figure** **2**a).

Meantime, the HCA/C showed the coincidental Li growth during further electrodeposition along with internal empty sites owing to facile charge transfer kinetics (Figure 2b). Therefore, the Li metal on the Cu electrode (Cu‐Li) exhibited a porous and lowly dense structure even under a low current density of 1 mA cm^−2^ as shown in Figure 2c. On the contrary, the Li metal in HCA/C (HCA/C‐Li) realized dense Li metal deposition inside the structure with negligible volume change compared with pristine HCA/C electrode (Figure 2d). The experimental results ensured that the HCA/C preserved the electrode volume during electrodeposition (Figure S7, Supporting Information). Porous and conductive structure could occupy consecutively deposited Li metal inside the structure compared with the Cu electrode to eventually thicken and make porous Li metal as more charges were induced.^[^ ^21^ ^]^

Besides, the HCA/C enabled fine Li densification under even harsh current density of 5 mA cm^−2^ without Li penetration out of the HCA owing to the as‐mentioned advantages however the Cu electrode resulted in a more problematic structure, hard to act as Li metal anode (Figure 2e, and f). With the effect of structural advantages, clear electrolyte wettability of HCA/C could additionally influence facile electrochemical kinetics to relatively reduce interface resistance (Figure S8, Supporting Information).

Unfortunately, all architectures prepared through NIPS technique with CB/SWCNT/PVA as structure ingredients cannot peremptorily facilitate positive effects to form smooth HCA/C‐Li anode. In this system, proper nonsolvent selection is one of the most important structure‐forming factors in designing hyperporous and hybrid conductors using complex. Thus, the undesirable nonsolvents (ethanol and 1‐butanol) showed distinct outcomes of uneven Li metal deposition due to inferior properties to construct the expected structures (Figure S9, Supporting Information).

### 2.3 Cycle Persistence of HCA/C‐Li Anode

**Figure 3:**
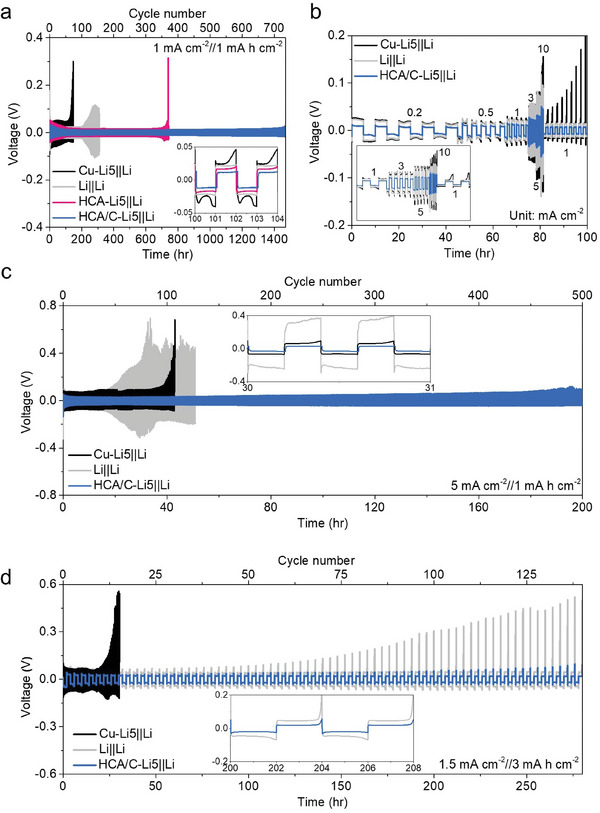
Cycle persistence of thin Li deposited electrodes. a) Cycling performance of asymmetric cells at a current density of 1 mA cm^−2^ with an areal capacity of 1 mAh cm^−2^. b) Rate durability of Cu‐Li5 and HCA/C‐Li5. Cycle efficiency under harsh electrochemical environments of Cu‐Li5||Li and HCA/C‐Li5||Li at c) the current density of 5 mA cm^−2^ with the areal capacity of 1 mAh cm^−2^ and d) the current density of 1.5 mA cm^−2^ with the areal capacity of 3 mAh cm^−2^.

Based on the kinetic difference in designing Li metal anode depending on the electrode structure, we confirmed that structures would directly affect electrochemical properties through asymmetric cell evaluation (**Figure** **3**). The asymmetric cell was composed of Li metal (thickness of 300 µm) was used as the reference/counter electrode where the Li metal (thickness of 40 µm), Cu‐Li5, HCA‐Li5, and HCA/C‐Li5 were selected as the working electrodes. Note that Li*x* (*x* = 5) indicates that the anode was adopted after electrochemical deposition of *x* mAh cm^−2^. Cu‐Li5||Li and Li||Li asymmetric cells experienced fast Li metal depletion and consecutive electrolyte consumption due to structure‐forming failure of Li metal anode, followed by continuous dendritic growth and Li isolation during cycling (Figure 3a).

On the other hand, while HCA‐Li5||Li and HCA/C‐Li||Li cells exhibited continuous deposition/dissolution cycles, the HCA‐Li|Li cell faced higher overpotentials and earlier cell failure, attributed to its lack of a robust backbone and lesser electronic connectivity compared to HCA/C. Notably, HCA/C‐Li anodes fabricated with ethanol and 1‐butanol were adversely affected, demonstrating a rapid lithium depletion at an early stage (Figure S10, Supporting Information). Particularly, the HCA/C fabricated by 1‐butanol showed much inferior electrochemical performance. This result is attributed to the low porosity of the host, indicating that only a minimal amount of Li can be integrated within the 3D host. Excess Li, having no space to deposit within the host, accumulates on its surface (top‐plating), thereby losing the intended advantages of the 3D host structure.

However, it is essential to highlight that the HCA/C‐Li anodes produced with 1‐propanol, our identified optimal condition, demonstrated exceptional electrochemical performance. This optimized structure enabled efficient modulation of Li‐ions and Li metal, facilitating prolonged cycle life and impressive fast‐charging capabilities. The optimized HCA/C‐Li||Li cells demonstrated significantly reduced electrochemical resistance even as current density was increased to 10 mA cm^−2^, without potential fluctuation, owing to the extensive electroactive sites facilitating simultaneous electrochemical reactions (Figure 3b). Furthermore, under fast‐charging conditions at a current density of 5 mA cm^−2^, these cells exhibited incomparable electrochemical performance compared to the Cu‐Li5||Li and Li||Li cells, which showed unstable reactions, higher overpotentials, and rapid degradation (Figure 3c).

To further elucidate the comparative performance, we also evaluated a traditional 3D Cu mesh under identical conditions for a direct comparison with our HCA/C electrodes (Figure S11, Supporting Information). The 3D Cu mesh‐Li5||Li cell demonstrated an escalation in overpotential from the 100^th^ cycle, ultimately reaching to severe Li depletion stage before the 250^th^ cycle. This outcome is primarily attributed to Li‐top plating in the 3D Cu mesh, a result of both the high electrical conductivity of the Cu mesh surface and the vertical electrical field present. This comparison starkly highlights the advanced capabilities of the HCA/C design in mitigating such challenges, offering a robust solution to the dendritic growth and overpotential issues commonly associated with traditional 3D Cu collectors.

To check the feasibility of full cell evaluation in advance, the asymmetric cell was evaluated under the increased areal capacity condition (1.5 mA cm^−2^//3 mAh cm^−2^) with a high Li utilization ratio (60%) in Figure 3d. Cu‐Li5||Li cell showed early cell failure before 20 cycles by higher Li metal utilization ratio to accelerate the depletion. Meantime, although Li||Li cell relatively persisted in prolonged cycles, it eventually faced a shortage of active Li metal by dead Li formation and consecutive SEI reformation along with a long tail of potential when the working electrode was under dissolution process. Whereas, HCA/C‐Li5||Li considerably delayed cell degradation to have the positive potential for practical full cell design.

### 2.4 Unveiling Electrochemical and Structural Strength of HCA/C

**Figure 4:**
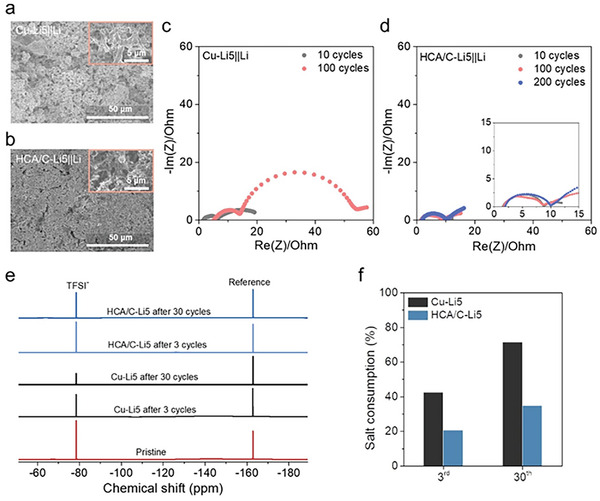
Post‐mortem analyses of thin Li deposited electrodes in cycled asymmetric cells. Top‐view SEM images of a) Cu from Cu‐Li5||Li and b) HCA from HCA/C‐Li5||Li5 after 30 cycles. Nyquist plots of c) Cu‐Li5||Li cell and d) HCA/C‐Li5||Li cell after different cycles. e) ^19^F NMR spectra of the electrolyte extracted from cells after selected cycles. f) The comparison ratio of salt consumption calculated from ^19^F NMR spectra after cycles.

To deeply understand the correlation between HCA/C structure and cycle persistence, various post‐morterm analyses were performed. As shown in **Figure** **4**a, dendritic Li structure in Cu‐Li5 remained after 30 cycles which indicates Li deposition with low densification and enormous dendrites continuously induced side reactions leading to fast Li and electrolyte depletion. Even though Li metal foil was adopted as an anode, it similarly showed porous and non‐uniform Li metal structure because raw Li cannot also control intrinsic electrochemical behavior without the supporting structure (Figure S12, Supporting Information).

In contrast, the HCA/C‐Li5 anode showed still densified structure without Li metal penetration out of the original HCA owing to the restraint of dendritic growth by lithiophilic properties of the complex (Figure 4b). The structural stability of the anode was also verified through time‐of‐flying secondary ion mass spectrometry (TOF‐SIMS) related to SEI endurance. Once the electrolyte was decomposed on the electrode, HCA/C‐Li5 sustained a stable organic layer on top and uniform distribution of inorganic compound and Li metal during cycling (Figures S13a, and b, Supporting Information). It indicates that the structure restrained excessive electrolyte decomposition and steadily maintained well‐penetrated ions for facile and repeatedly dense Li electrodeposition.

However, the Cu‐Li5 anode showed non‐uniform electrolyte decomposition, evidenced by poor distribution of ionized fluorine and sulfur and loosely packed Li^−^ at the electrode surface (Figures S13c, and d, Supporting Information). The degradation cause of Cu‐Li5 anode was also detected in electrochemical impedance spectroscopy (EIS) after selected cycles (Figure 4c). As the cycle proceeded, the cell displayed higher bulk resistance, affected by continuous Li salt consumption for SEI reconstruction. Besides, the thickening SEI layer and poor anode densification influenced cell resistance increase and inferior properties in the fast‐charging system. In the case of HCA/C‐Li5 anode considerably maintained the cell environment well for 200 cycles with negligible side reaction and resistance increase (Figure 4d).

The fatal destruction of electrolytes was obviously unveiled by fluorine nuclear magnetic resonance (^19^F NMR) analysis against undesirable Li deposition/dissolution process on cycling (Figure 4e). To quantify the changes in the electrolyte amount, the quantity of bis(trifluoromethane sulfonyl)imide (TFSI^−^) anions in the cell was tracked since F signals come exclusively from the LiFSI salt (see details in the Experimental Section). Extracted electrolyte from cells after selected cycles provides the change of salt concentration by comparing with a known amount of an internal reference (hexafluorobenzene, −162.8 ppm in the ^19^F NMR spectra).^[^ ^22^ ^]^ As salt consumption was calculated, the Cu‐Li5 anode distinctly spent lots of Li salt due to SEI reformation by uncontrollable behavior of Li metal where the salt was consumed almost twice rather than HCA/C‐Li5 after 30 cycles (Figure 4f).^[^ ^23^ ^]^

### 2.5 Full Cell Evaluation with Diverse Cathodes

In designing a full cell, the porous architecture as a Li metal host typically has few restraints in the aspect of N/P ratio consideration because the HCA/C‐Li cannot be infinitely receptive against Li metal deposition in the limited pore volume of the structure. Excessive Li deposition in this system induces Li metal penetration and new interphase formation on top of HCA and therefore the factor would interrupt efficient cell building of full cells (Figure S14, Supporting Information). In this regard, it is important to easily design the HCA/C tailoring the amounts of Li metal deposition and the strong advantage of our synthetic system is to easily modify the electrode thickness in the electrode fabrication process (Figure S15, Supporting Information). Thus, we diversify HCA/C electrodes depending on cell design requirements and applications.

**Figure 5:**
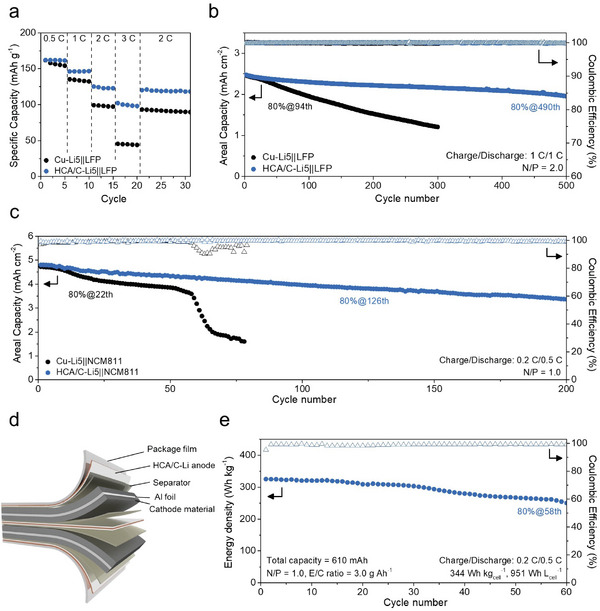
Electrochemical evaluation of full cells. a) Rate performance and b) Long‐term cycle persistence of Cu‐Li5||LFP and HCA/C‐Li5||LFP, assembled using coin cells, with N/P ratio of 2.0 (1 C = 2.5 mA cm^−2^). c) Cycle retention of Cu‐Li5||NCM811 and HCA/C‐Li5||NCM811 coin cell with high‐loaded cathode and N/P ratio of 1.0 (1 C = 5.0 mA cm^−2^). d) Schematic illustration to elucidate the configuration inside the designed stack pouch full cell. This practical pouch cell was prepared with an N/P ratio of 1.0 and limited amounts of liquid electrolyte (3.0 g Ah^−1^). e) Cycle performance of the pouch cell (1 C = 20 mA cm^−2^).

Based on the improved electrochemical and structural strength of HCA/C, the various full cell configuration was evaluated to confirm the feasibility of practical LMB application (**Figure** **5**). Here, we used the same HCA/C thickness, adopted to asymmetric cell evaluation to unify anode condition. At first, LiFePO_4_ (LFP) cathode was paired with Cu‐Li5 and HCA/C‐Li5 anode, respectively. LFP cathode featuring an areal capacity of 2.5 mAh cm^−2^ was prepared to design an N/P ratio of 2.0 and realized a limited Li metal environment. Both cells experienced similar formation cycles in Figure S16 (Supporting Information) however owing to the competitiveness of fast‐charging conditions, HCA/C‐L5||LFP exhibited considerable capacity retention of 63.1% at 3C against 0.5C while Cu‐Li5||LFP is hard to endure fast electrochemical condition which showed only capacity retention of 28.5% at the same ratio of current density (Figure 5a). Further, HCA/C‐Li5||LFP showed a much more stable cycle ability to maintain the capacity retention of 80% to 490 cycles rather than Cu‐Li5||LFP at 1C (2.5 mA cm^−2^) which reached that retention before 100 cycles (Figure 5b). HCA/C could enable to support of a sturdy anode integrity even in full cell evaluation.

Despite the remarkable cycle stability and the advantageous low N/P ratio in the HCA/C‐Li||LFP system, the cell exhibits a relatively low energy density, primarily attributed to the intrinsic limitations of the LFP cathode – specifically, its low specific capacity (150 mAh g^−1^) and working voltage (3.3 V vs Li/Li^+^).^[^ ^24^ ^]^ To realize a high‐energy‐density battery application, LiNi_0.8_Co_0.1_Mn_0.1_O_2_ (NCM811) cathode and high‐voltage ether‐based electrolyte, capable of delivering a higher specific capacity of 200 mAh g^−1^ and an elevated working voltage of 3.8 V vs Li/Li^+^.^[^ ^25^ ^]^ NCM811 cathode was prepared with two kinds of competitive loading levels (8.0 and 23.4 mg cm^−2^) and areal capacity (1.7 and 5.0 mAh cm^−2^) along with N/P ratios of 3.0 and 1.0 to separately elucidate quantitative electrochemical behavior and practical battery application. For formation cycle, Cu‐Li5||NCM811 and HCA/C‐Li5||NCM811 showed similar results without remarkable electrochemical properties at 0.1C (1C = 1.7 mA cm^−2^) in Figure S17 (Supporting Information).

However, Cu‐Li5||NCM811 displayed fast decay of capacity, reaching the capacity retention of 80% at the 128^th^ cycle while HCA‐C/Li5||NCM811 sustained robust cycle life over 400 cycles with a capacity retention of 77.4% (Figure S18, Supporting Information). Even though Li metal foil (thickness of 40 µm) was applied to exclude the loosely packed electrode structure of deposited Li anode (Cu‐Li), it cannot widely extend cycle life compared with Cu‐Li5, showing 80% capacity reaching after only 146 cycles (Figure S19, Supporting Information). Besides, Cu‐Li5||NCM811 suffered from a large potential gap and severe IR drop as the cycle proceeded, attributed to the reduction of ion mobility and poor kinetics for charge transfer (Figure S20a, Supporting Information).^[^ ^26^ ^]^ Whereas, the HCA/C‐Li5||NCM811 relatively stabilized electrochemical behavior for 200 cycles, confirming the HCA/C ensured the reversibility of Li metal without serious side reaction and enabled to extend battery cycle life (Figure S20b, Supporting Information).

### 2.6 Practical Feasibility of HCA/C‐Li||NCM811 Full Cell

These differences were dramatically verified with a thick NCM811 cathode with a remarkable areal capacity of 5 mAh cm^−2^ and a low N/P ratio of 1.0. Cu‐Li5||NCM811 experienced a sudden drop of the capacity during cycling at 0.2C/0.5C (charge/discharge, 1C = 5 mA cm^−2^) related to Li metal and/or electrolyte depletion by high utilization of deposited Li metal in the anode (Figure 5c). In contrast, HCA/C‐Li5||NCM811 maintained the outstanding cycle retention (80% at 125^th^ cycle) and endured cycle operation without cell failure. This result demonstrated the outstanding electrochemical performance of HCA/C electrode even in a very low N/P ratio of 1.0 and high areal capacity which suggested the feasibility of high‐energy‐density LMBs even under these rigorous conditions, outperforming the previously reported works related to 3D host for LMAs (Table S2, Supporting Information).^[^ ^27^ ^]^

We also conducted post‐mortem analyses of HCA/C‐Li and Cu‐Li from the full cells after 70 cycles (Figure S21, Supporting Information). The analyses revealed that HCA/C‐Li showed dense surface morphology without any noticeable Li dendrite, demonstrating the Li‐filling ability to suppress Li dendrite. In contrast, the Cu‐Li counterpart exhibited a markedly porous surface characterized by the extensive formation of lithium dendrites. This result is well‐consistent with the post‐mortem analyses from the asymmetric cells in Figures 4a and b. Besides, the HCA/C electrode showed the feasibility to ultimately achieve the full cell even as a Li‐free anode where other types of HCA/C||Li asymmetric cell and HCA/C||NCM811 full cell along with full Li utilization exhibited higher cycle efficiency and stable cycle retention (Figure S22, Supporting Information).

By extension, we fabricated stacked pouch full cells to realize the ultimate practical system and high‐energy‐density LMBs. HCA/C has an additional attractive point in terms of the building of cell design because the weight of subsidiary materials should be reduced to manufacture a single battery cell with high energy density. In this regard, all carbon‐based HCA/C electrode features intrinsically low density and the experimental result corroborates that the electrode showed ultra‐lightweight (6.16 mg), compared with the Cu electrode (32.2 mg) for the same area (Figure S23, Supporting Information). With the comprehensive advantages, the HCA/C‐Li5||NCM811 stacked pouch cell was assembled as illustrated in Figure 5d and the HCA/C electrode substantially reduced the weight of anode where the HCA/C occupied the weight ratio of 4.2% compared with Cu (that of 19.3%) in total weight of pouch cell (Figure S24 and Table S3, Supporting Information).

In terms of cell operation, the cycling efficiency of LMBs at such a low pressure was poor due to the growing amounts of dendritic Li metal deposition because the morphology of LMA is significantly influenced by physical pressure from cell zig.^[^ ^28^ ^]^ Thus, The pressure, applied to the stacked pouch cell, was constrained at a low pressure of ≈20 kPa to ultimately confirm the Li densification ability of HCA/C on cycling in practical application The assembled pouch cell exhibited the capacity of 0.61 Ah with stable electrochemical behavior at the formation cycle (Figure S25, Supporting Information) and besides the cell realized the gravimetric energy density of 344 Wh kg_cell_^−1^ and volumetric energy density of 951 Wh L_cell_^−1^ as calculated including all cell components, not partially considered in the limitation to electrodes (Figure S26 and Table S4, Supporting Information). Such a high energy density could be achieved through the ultra‐lightweight anode and very low N/P ratio of 1.0 under a limited amount of electrolytes (electrolyte mass/cell capacity (E/C) ratio of 3.0 g Ah^−1^).

This specification is quite comparable to as‐reported practical LMB pouch cells even though fewer stack layers were adopted in our pouch cell (Table S5).^[^ ^29^ ^]^ We expect that the cell energy density can be further improved by increasing the number of stack configurations that will enable the reduction of the weight/volume portion of package substances in the pouch full cells. Meantime, the HCA/C‐Li5||NCM811 pouch cell displayed an energy density retention of 76.9% after 60 cycles even under such a low pressure (≈20 kPa), attributed to the excellent Li‐absorbing capability of HCA/C (Figure 5e). These competitive results not only reinforce the practicality of our newly designed electrodes in fabricating stacked pouch full cells but also mark a significant stride towards realizing high‐energy‐density LMBs. Besides, the key to this achievement lies in the rational design of the ultra‐lightweight electrode, combined with the strategic densification of Li metal as a capable Li metal anode, heralding a new era in battery electrode structure.

## 3 Conclusion

In summary, we have developed an innovative LMA model, employing a hyperporous/hybrid conductive architecture on SWCNT film (HCA/C) through the NIPS process. This architecture serves dual roles as both a Li host and a current collector. By integrating ionic polymers with conductive carbon materials, our approach marks a significant improvement over traditional Cu or stainless‐steel foils, notably reducing the weight of the LMA and contributing to the practical achievement of high‐energy‐density battery systems with stable electrodeposition/dissolution operations.

The HCA/C has not only allowed for facile fabrication and fine control over pore structures but also significantly boosted the electrochemical performance and energy density of LMBs. It enables the deposition of uniform and dense Li within its structure even under fast charging conditions (up to 5 mA cm^−2^), with low nucleation and growth potential, attributed to the lithiophilic and electrical interconnection pathway. The asymmetric cell incorporating HCA/C shows stable electrochemical performance and prolonged cycling life, effectively mitigating consecutive Li and electrolyte consumption. In the full cell test, the HCA/C‐Li5||LFP with an areal capacity of 2.5 mAh cm^−2^ and an N/P ratio of 2 retained 80% of its initial capacity after 490 cycles.

Furthermore, the HCA/C‐Li5||NCM811 full cell, boasting a high areal capacity (5 mAh cm^−2^) and a low N/P ratio (1.0), demonstrated stable cycling performance over 200 cycles. Importantly, we have fabricated a stacked pouch‐type full cell to realize high‐energy‐density LMBs, which achieved an energy density of 344 Wh kg^−1^_cell_/951 Wh L^−1^_cell_, including all cell components, surpassing previously reported pouch‐type full cells. The stacked pouch‐type full cell not only represents a significant technical breakthrough in battery research but also offers a scalable and practical solution for the widespread adoption of high‐energy‐density batteries.

By addressing the critical aspects of lithium metal anode design, such as the stabilization of lithium deposition and the enhancement of electrochemical kinetics, this study provides a new avenue for the development of stable LMAs for high‐energy‐density LMBs.

## 4 Experimental Section

### Materials

PVA (molecular weight = 31–50 kDa) was purchased from Sigma‐Aldrich. SWCNT aqueous solution (0.2 wt% in DI water) was purchased from KORBON Co. Super P carbon black was purchased from Imerys. Ethanol, 1‐propanol, and 1‐butanol were purchased from SAMCHUN Chemicals. LFP powder was purchased from Süd Chemie and NCM811 powder was purchased from POSCO Future M. PVDF (KF1100, *M*_n_ = 168.8 kDa, polydispersity index = 2.94, KUREHA Chem. Ind.) was used as the polymeric binder in the cathode electrode.

### Fabrication of HCA

SWCNT film was fabricated via vacuum filtration of SWCNT aqueous solution. PVA of 0.4 g and 0.33 g of super P were added to 4.6 g of SWCNT solution and vigorously stirred at 90 °C for 3 hr. The homogeneously mixed solution was cast on a SWCNT film and then immediately immersed in the nonsolvent bath for 6 hr at room temperature. After sufficient solvent‐nonsolvent exchange, the 3D host was dried in a vacuum oven at 70 °C for 3 hr to remove the solvent.

### Characterization

Scanning electron microscopy was used to obtain the morphological structures (SEM, S‐4800, Hitachi). Cary 600 was used to collect the FT–IR spectra (Agilent Technologies). Droplet Analyzer (Smart Drop) contact angle measurements were performed to observe the affiliation between HCA and the electrolyte. The porosity and pore size distribution were analyzed by a mercury porosimeter (Micromeritics, AutoPore IV 9500). Time‐of‐flight secondary ion mass spectrometry (TOF‐SIMS) 3D profiling and depth analysis was conducted with TOF‐SIMS 5, ION TOF.

### Electrochemical Measurements

The electrolyte‐impregnated ionic conductivity was measured using coin cells, which were built with two blocking stainless‐steel (SS) blocking electrodes (16 mm in diameter) separated by different electrodes and the electrolyte was filled. The electrolyte‐impregnated ionic conductivity was calculated by Equation 2.

(2) $$\sigma \;=\frac{L}{R_{b0}\times S}\;$$

where *σ* was the ionic conductivity, *R_bo_* was the bulk resistance, and *L* was the thickness between two SS blocking electrodes. The electronic conductivity was measured by applying a direct current (5 mA) to the cells assembled with different substrates sandwiched between two SS blocking electrodes. The electronic conductivity was calculated using Equation 3.

(3) $$\rho \;=\frac{1}{\sigma_{e}}\;=\frac{R\times S}{L}\;=\frac{U\times S}{I\times L}\;$$

where *ρ* was the electrical resistivity, *σ_e_* was the electronic conductivity, *U* was the average voltage increase, *I* was the applied current of 5 mA, *S* was the contact area between SS and anode, and L was the anode thickness. LFP and NCM811 cathodes were prepared by mixing super P and PVDF binder in a mass ratio of 90:5:5 and 95:2:3, respectively, and casting the slurries on aluminum foil (18 µm). The mass loading of LFP was 16.7 mg cm^−2^. For the NCM811 cathode, two kinds of mass loading (8.0 and 23.4 mg cm^−2^) were prepared to confirm both general electrochemical properties and practical application. CR2032‐type coin cells were assembled to investigate the electrochemical performances of the asymmetric and full cells in an Argon‐filled glove box. Celgard 2400 was employed as the separator. Electrolyte dissolving 1.0 m lithium bis(trifluoromethanesulfonyl)imide (LiTFSI) in 1,3‐dioxolane (DOL)/1,2‐dimethoxyethane (DME) (v/v = 1/1) with 2 wt% LiNO_3_ for was used for asymmetric and full cells, paired with LFP cathode. Electrolyte dissolving 2.0 m LiFSI in DME with 1 wt% lithium difluoro(bisoxalato)phosphate (LiDFBP) and 3 wt% LiNO_3_ was used for NCM811 full cells to operate cell test in wide a potential range to 4.2 V (vs Li/Li^+^). To fabricate HCA‐Li5 and Cu‐Li5, 5 mAh cm^−2^ of Li metal was electrodeposited on HCA/C and Cu electrodes before assembling asymmetric and full cells. The full cells with LFP cathodes were evaluated in the potential window of 2.8–3.8 V (versus Li/Li^+^). The full cells with NCM811 were tested in the potential window of 2.7–4.2 V (vs Li/Li^+^) using constant current/voltage (CC/CV) mode at 0.05 C on charge state. The EIS measurements were conducted between 100 kHz and 0.1 Hz by potentiostatic EIS with an amplitude of 10 mV (VSP‐300, BioLogic).

### Quantifying Electrolyte Retention

The ^19^F NMR test was conducted to quantify the electrolyte retention during cycling. The electrolyte was extracted from the cycled cells with 0.3 mL of dimethyl carbonate. The solution was then mixed with 0.3 mL of deuterated dimethylsulfoxide solution containing 1 wt% hexafluoribenzene in an NMR tube. The same procedure was followed for preparing uncycled electrolyte samples to minimize errors. The electrolyte amount, corresponding to the peak area of TFSI anions, was normalized to 100% by comparing it with the internal reference (hexafluoribenzene). This approach allows to quantitatively measure the electrolyte retention during cycling due to the constant content of the internal reference in all samples.

### Pouch Cell Fabrication

The stacked pouch‐type cell (composed of HCA‐Li5 and NCM811 cathode) was fabricated in a dry room, managed with a dew point of ‐50 °C. Further, for double‐sided anode realization, HCA/C/HCA electrode configuration was prepared through individual NIPS processes on both sides (Figure S27, Supporting Information). The cell was built with the dimensions of 50 mm × 60 mm (cathode), 53 mm × 63 mm (anode), and 56 mm × 66 mm (separator). The tolerance of electrodes was quite largely designed to exclude the factor related to cell fabrication. The total capacity of the stacked pouch‐type cell was ≈ 0.61 Ah, consisting of two double‐sided cathodes, one double‐sided, and two single‐sided HCA/C‐Li5. Therefore, the stacked pouch‐type cell was designed with the N/P ratio of 1.0 and the amount of the electrolyte, used in the pouch cell, was 1.8 g, corresponding to an E/C ratio of 3.0 g Ah^−1^. The electrochemical performance of the stacked pouch cell was evaluated after initial formation for 2 cycles in the potential window of 2.7–4.2 V at 25 °C at 0.1C for both charge/discharge (1C = 20 mA cm^−2^). The prolonged cycle test was evaluated at 0.2C/0.5C for charge/discharge and the voltage range of 2.7–4.2 V at 25 °C under the presence of external pressure (20 kPa). The information of cell parameters of the pouch cell was summarized in Table S3 (Supporting Information). Calculation details for gravimetric/volumetric energy densities of the fully packaged cell (including all cell components) were described in Table S4(Supporting Information).

(4) $$\begin{array}{ccc} & & \mathrm{Gravimetric}\;\mathrm{energy}\;\mathrm{density}\;\left(\mathrm{Wh}\;kg^{-1}\right)\\ & & =\frac{\mathrm{Capcity}\;\left(\mathrm{Ah}\right)\times \mathrm{working}\;\mathrm{voltage}\;\left(V\right)}{\mathrm{Total}\;\mathrm{weight}\;\mathrm{of}\;\mathrm{pouch}\;\mathrm{cell}\;\left(\mathrm{kg}\right)}\; \end{array}$$

(5) $$\begin{array}{ccc} & & \mathrm{Volumetric}\;\mathrm{energy}\;\mathrm{density}\;\left(\mathrm{Wh}\;L^{-1}\right)\\ & & =\frac{\mathrm{Capcity}\;\left(\mathrm{Ah}\right)\times \mathrm{working}\;\mathrm{voltage}\;\left(V\right)}{\mathrm{Total}\;\mathrm{volume}\;\mathrm{of}\;\mathrm{pouch}\;\mathrm{cell}\;\left(L\right)}\; \end{array}$$

## Conflict of Interest

The authors declare no conflict of interest.

## Supporting information

Supporting Information

## Data Availability

The data that support the findings of this study are available from the corresponding author upon reasonable request.
