# Supplementary material for: Facile Lithium Densification Kinetics by Hyperporous/Hybrid Conductor for High‐Energy‐Density Lithium Metal Batteries
Source: Adv Sci (Weinh). 2024 Apr 22;11(25):2402156. doi: 10.1002/advs.202402156 (PMC11220661; doi:10.1002/advs.202402156)
Supplement: Supplementary file 1 — Supporting Information [file ADVS-11-2402156-s001.pdf]

ADVANCED SCIENCE
Open Access

Supporting Information

for *Adv. Sci.*, DOI 10.1002/advs.202402156

Facile Lithium Densification Kinetics by Hyperporous/Hybrid Conductor for High-Energy-Density Lithium Metal Batteries

Dong-Yeob Han, Saehun Kim, Seoha Nam, Gayoung Lee, Hongyeul Bae, Jin Hong Kim, Nam-Soon Choi, Gyujin Song and Soojin Park**

Supporting Information

Facile Lithium Densification Kinetics by Hyperporous/Hybrid Conductor for High-Energy-Density Lithium Metal Batteries

Dong-Yeob Han, Saehun Kim, Seoha Nam, Gayoung Lee, Hongyeul Bae, Jin Hong Kim, Nam-Soon Choi, Gyujin Song, Soojin Park**

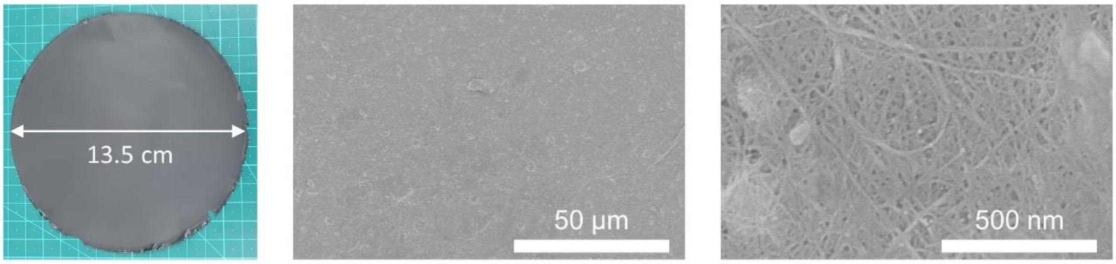

Figure S1. (a) A photograph and (b) SEM images of as-prepared CNT film.

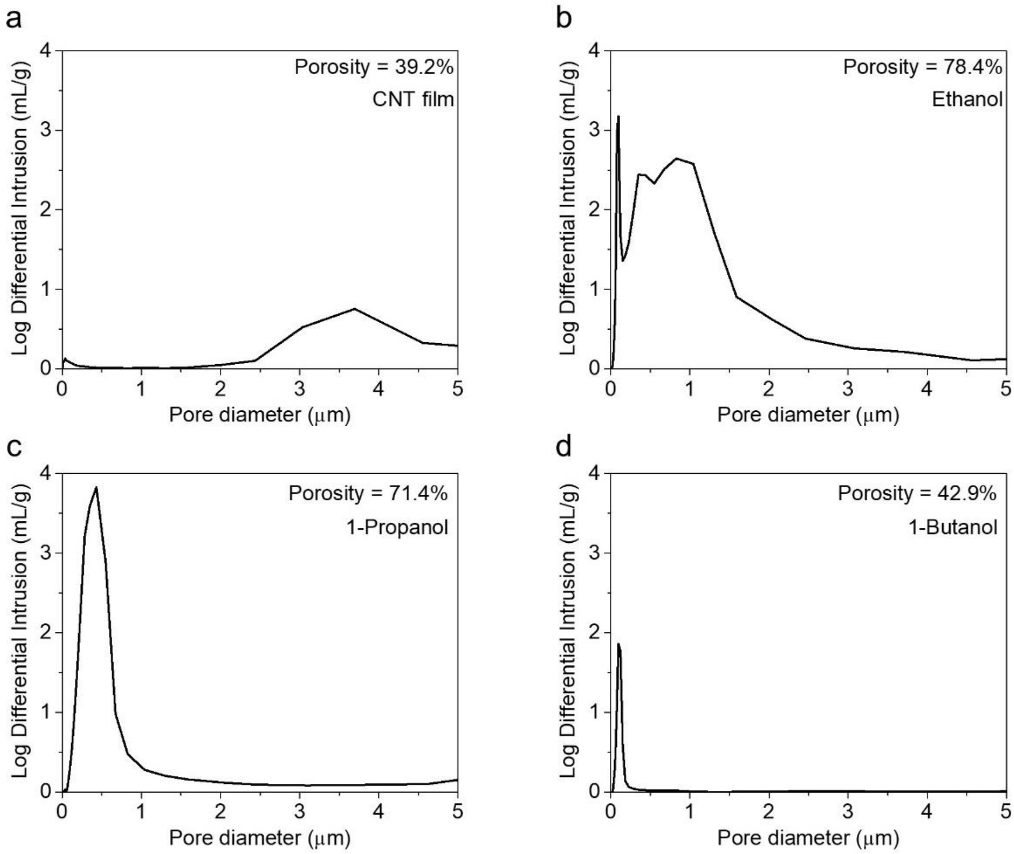

Figure S2. Mercury intrusion porosimetry plots showing pore size distribution of (a) CNT film and fabricated HCA with (b) ethanol, (c) 1-propoanol, and (d) 1-butanol as a nonsolvent.

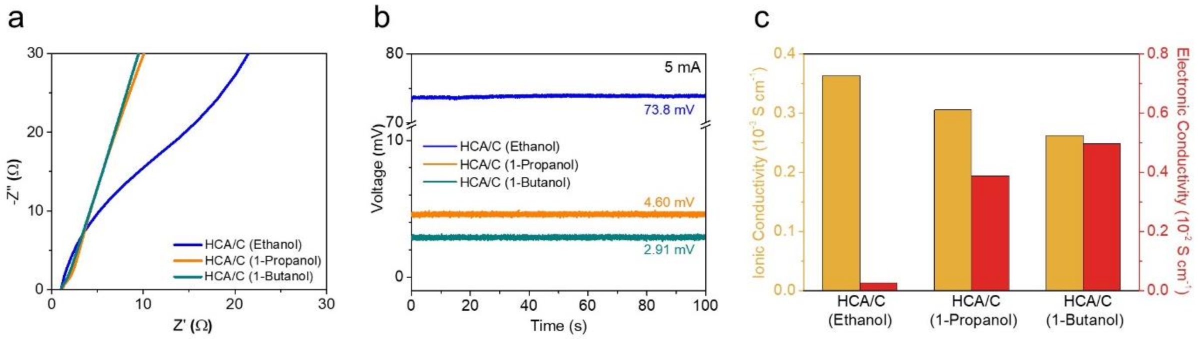

Figure S3. (a) Nyquist plots of various HCA/C with different nonsolvent, soaked in the electrolyte. (b) The voltage response of different substrates to 5 mA applied current using stainless steel (SS) blocking electrodes. (c) Comparisons of electronic conductivity and electrolyte-impregnated ionic conductivity for various HCA/C with different nonsolvent.

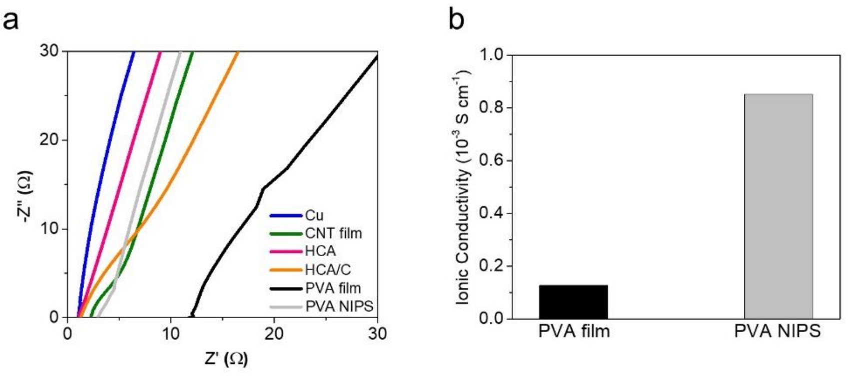

Figure S4. (a) Nyquist plots comparison of various electrode, soaked in the electrolyte with blocking cell. (b) electrolyte-impregnated ionic conductivity of PVA polymer film by spin coating process and PVA architecture by NIPS process

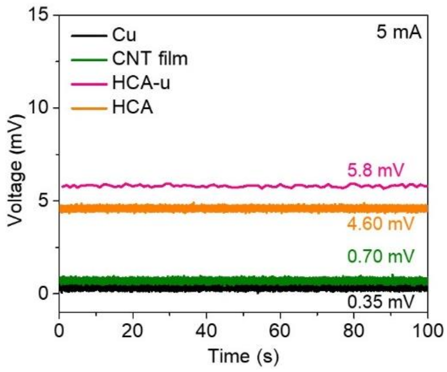

Figure S5. (a) The voltage response of different substrates to 5 mA applied current using stainless steel (SS) blocked electrodes.

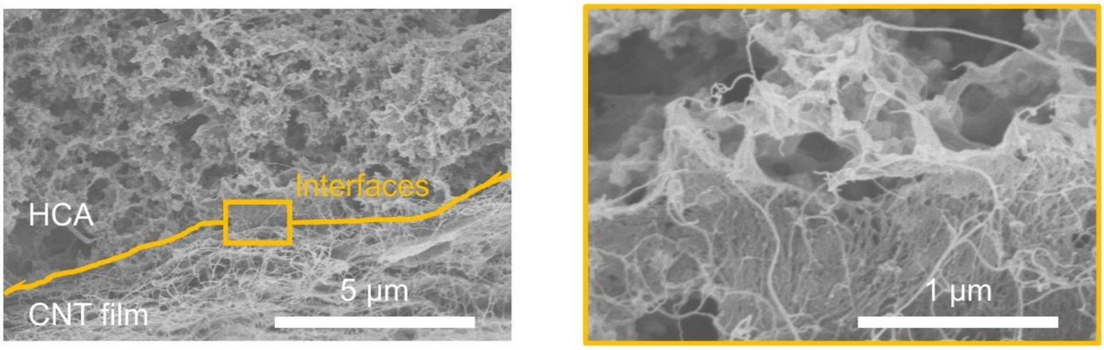

Figure S6. SEM images of an interface between HCA/C and CNT film.

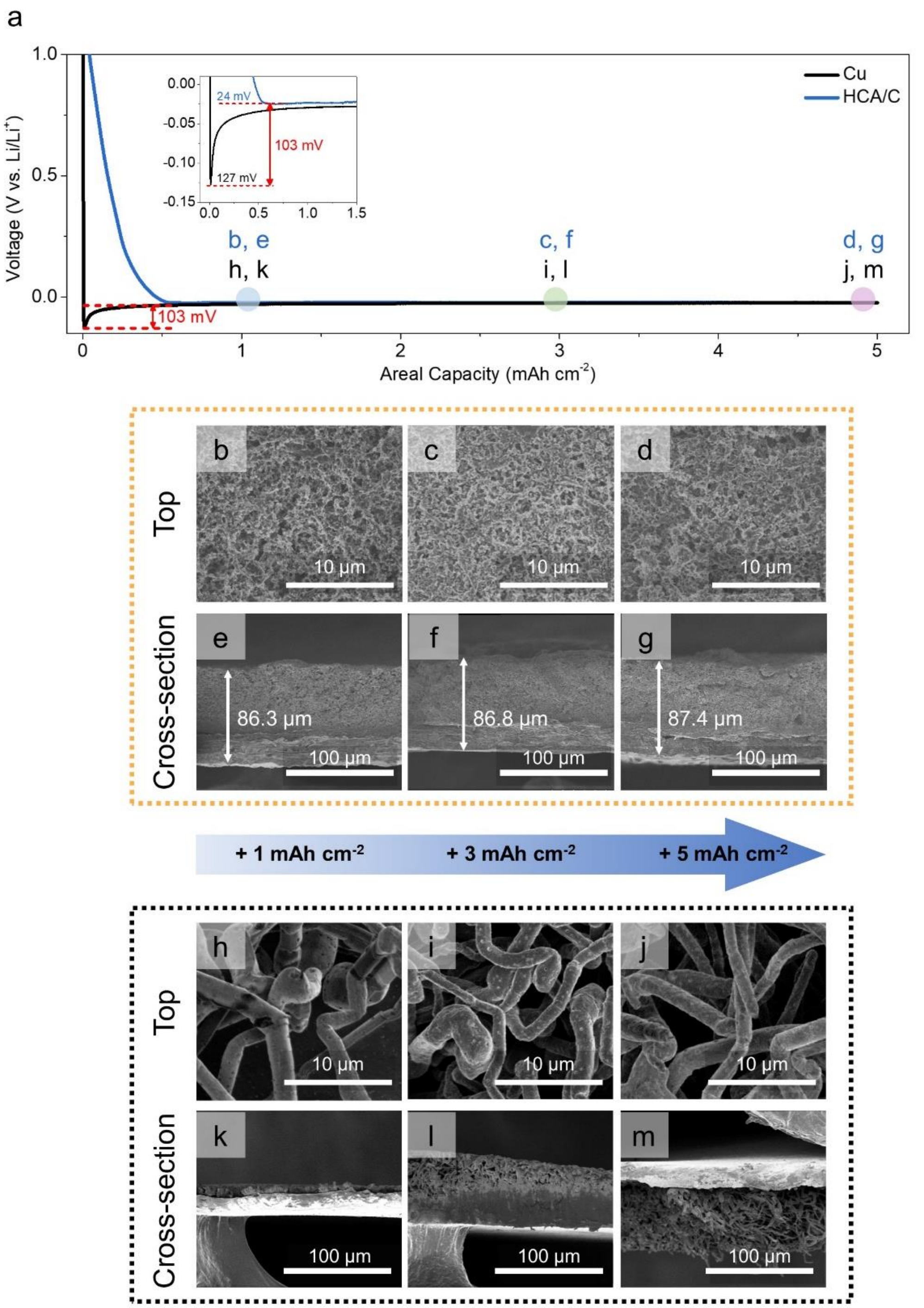

Figure S7. (a) Voltage profiles of lithium electrodeposition on Cu and in HCA/C until reaching to the areal capacity of 5 mAh cm^{-2}. SEM images of (b-g) HCA/C and (h-m) Cu after plating of (b, e, h, k) 1 mAh cm^{-2}, (c, f, i, l) 3 mAh cm^{-2}, and (d, g, j, m) 5mAh cm^{-2} of deposited Li under the current density of 0.5 mA cm^{-2}.

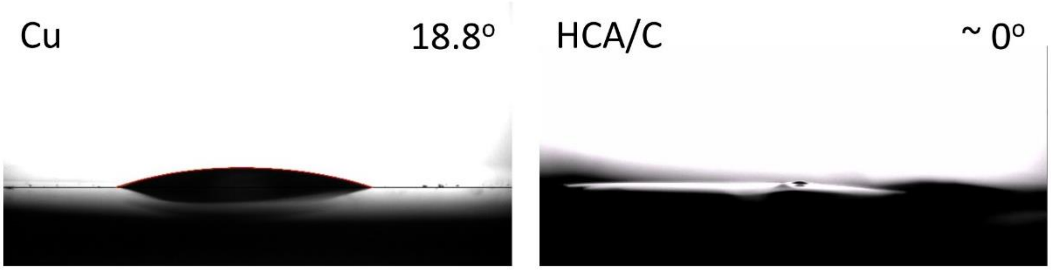

Figure S8. Contact angle observation just after physical contact between of electrodes (Cu and HCA/C) and electrolyte droplet to confirm the comparison of electrolyte impregnation.

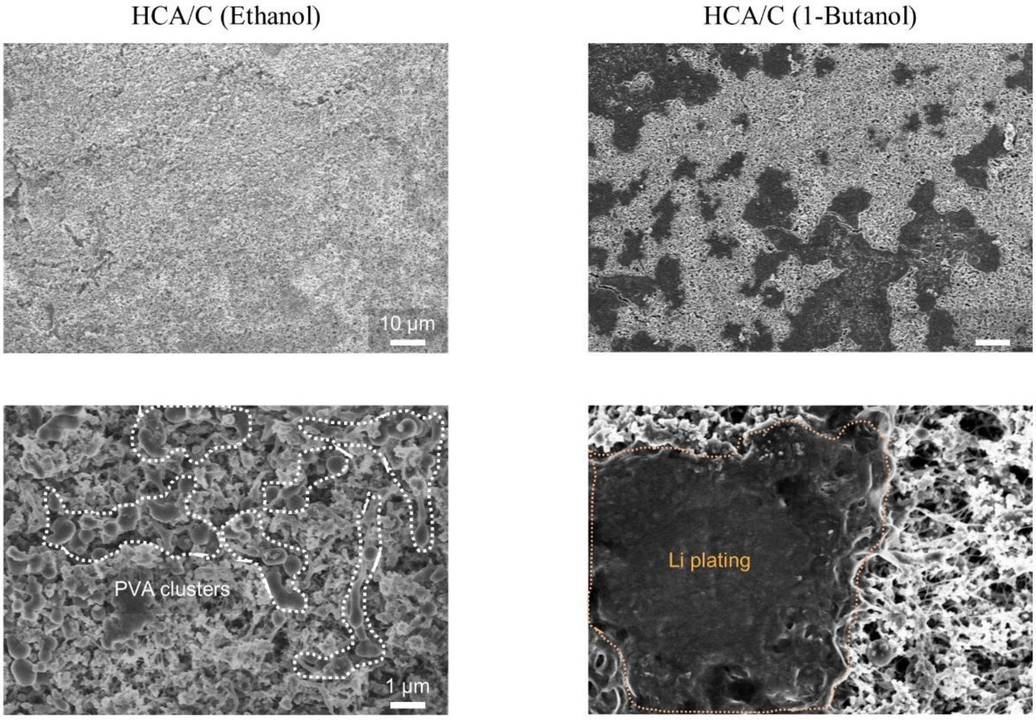

Figure S9. Top-view SEM images of HCA/C, constructed using other nonsolvents (ethanol and 1-butanol) after electrodeposition of 5 mAh cm^{-2}.

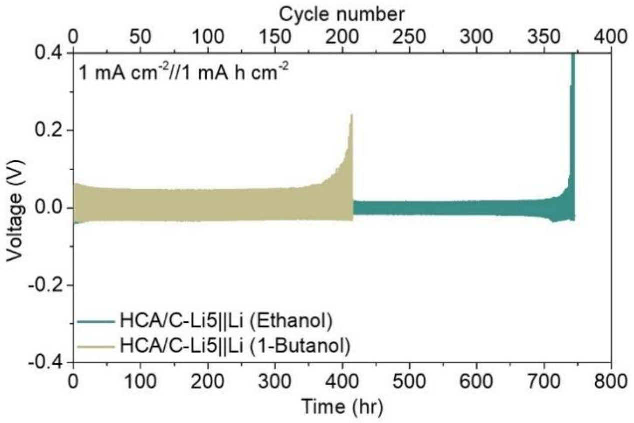

Figure S10. Cycle persistence of asymmetric cells (HCA/C-Li5||Li) fabricated by other nonsolvents (ethanol and 1-butanol).

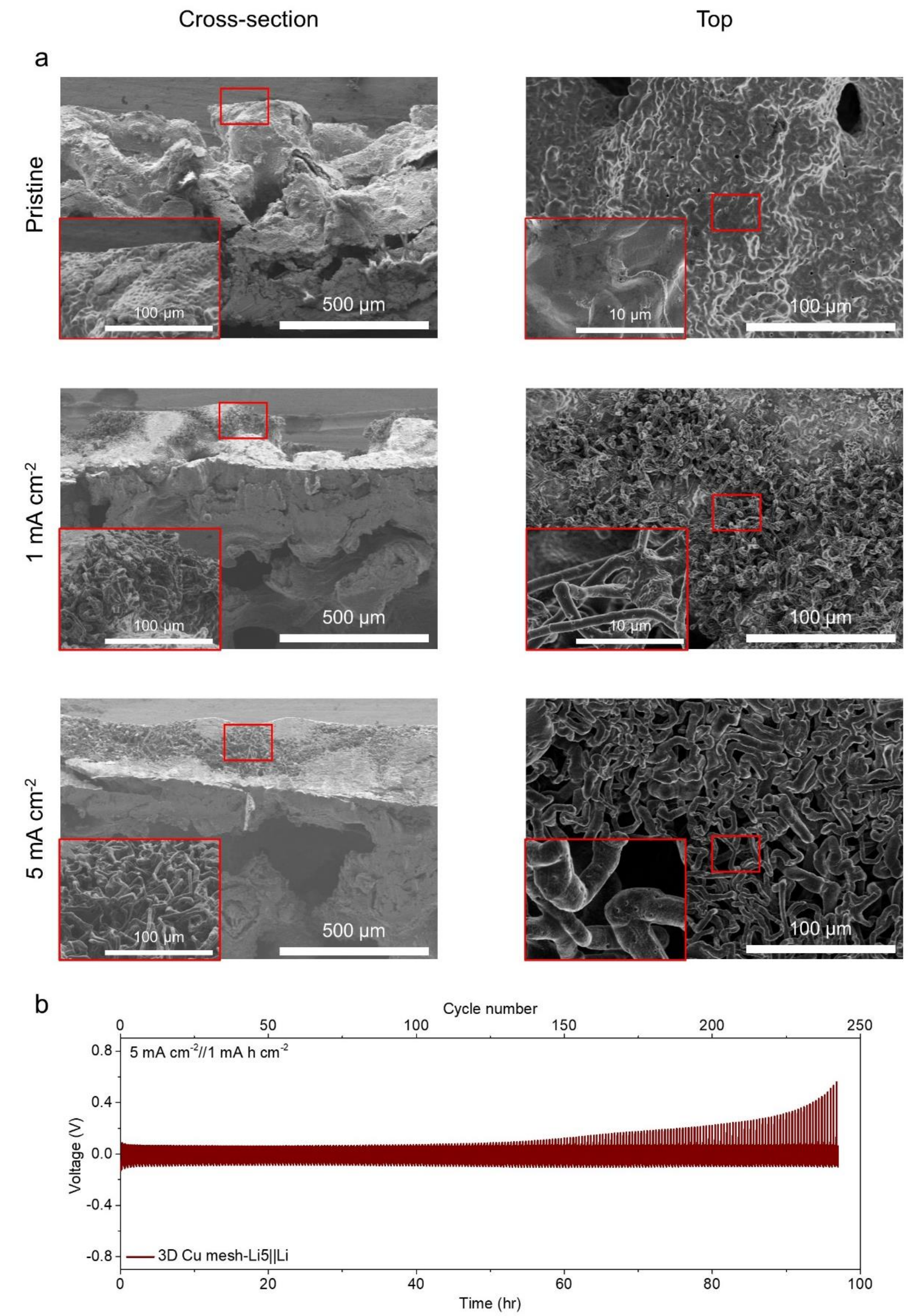

Figure S11. (a) SEM images before and after lithium deposition of 5 mAh cm^{-2} on 3D Cu mesh under current densities of 1 mA cm^{-2} and 5 mA cm^{-2}. (b) Cycling performance of an asymmetric cell with 3D Cu mesh at high current density of 5 mA cm^{-2}.

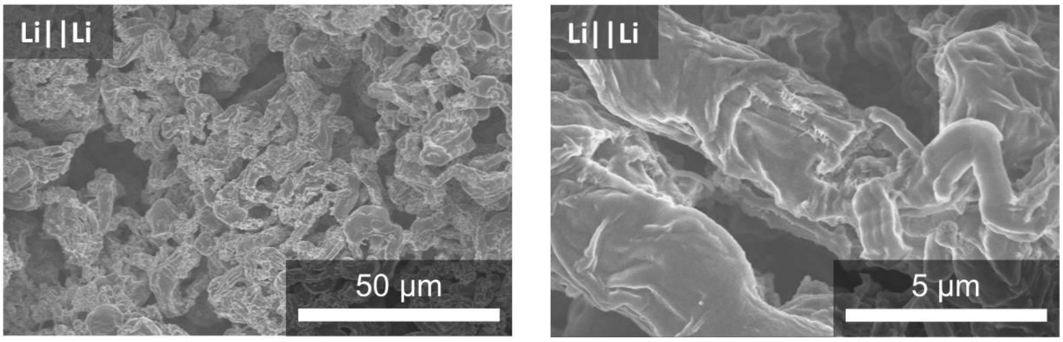

Figure S12. Top-view SEM images of Li||Li after 30 cycles.

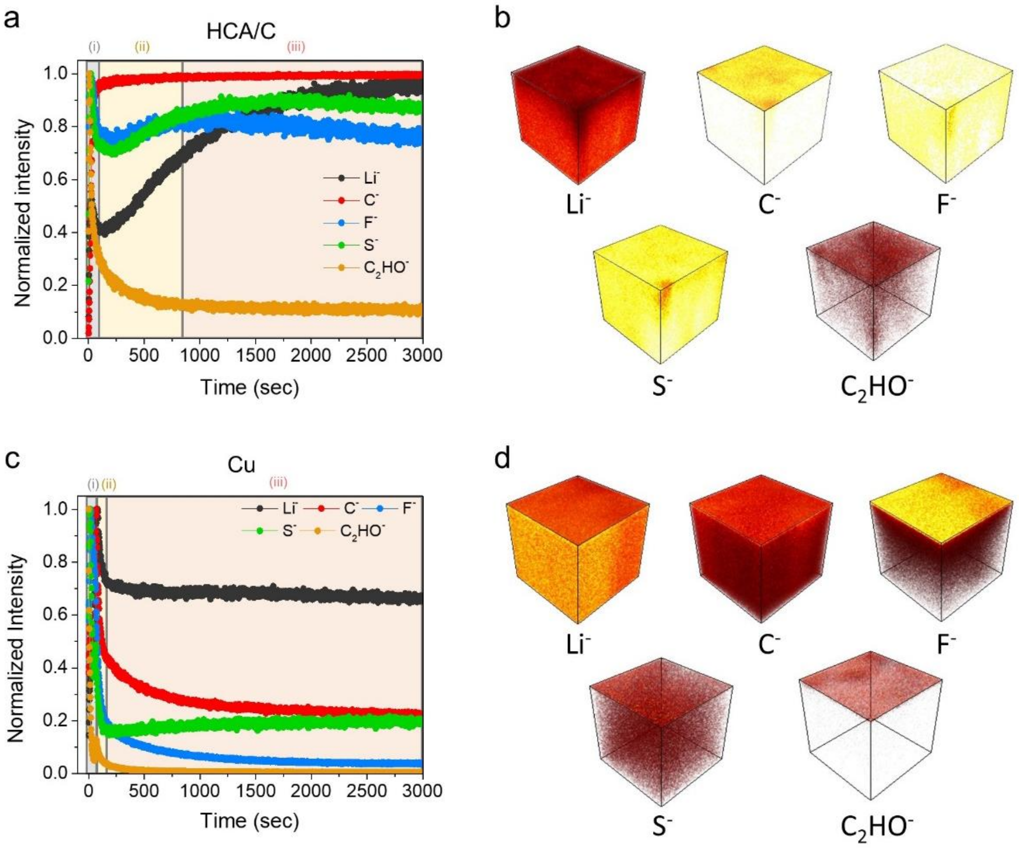

Figure S13. Normalized TOF-SIMS depth profiles of characteristic fragments sputtered from (a) HCA/C and (c) Cu after 30 cycles. 3D variation of the TOF-SIMS intensity related to the corresponding characteristic fragments from (b) HCA/C and (d) Cu after 30 cycles. ((i) Organic SEI layer, (ii) Inorganic SEI layer & Li metal, and (iii) Substrate or subsrate-Li metal)

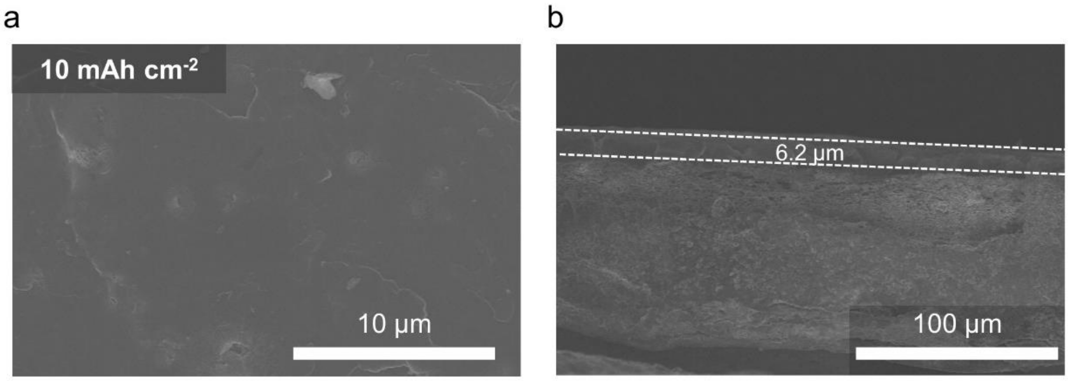

Figure S14. (a) Top- and (b) cross-sectional-view SEM images of HCA/C after over-plating of 10 mAh cm^{-2} Li under the current density of 0.5 mA cm^{-2}.

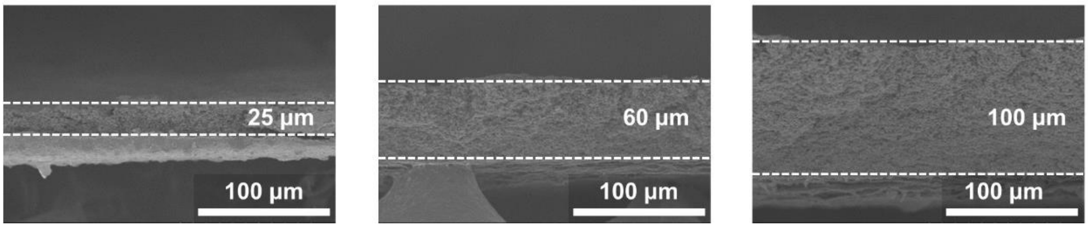

Figure S15. SEM images of HCA/C to verify the feasibility of thickness control depending on the amounts of lithium accommodation in HCA.

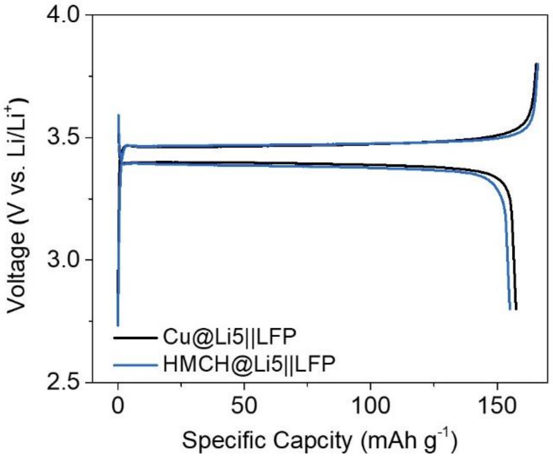

Figure S16. Galvanostatic voltage profiles of Cu-Li5||LPF and HCA/C-Li5||LFP. (N/P ratio of 2.0)

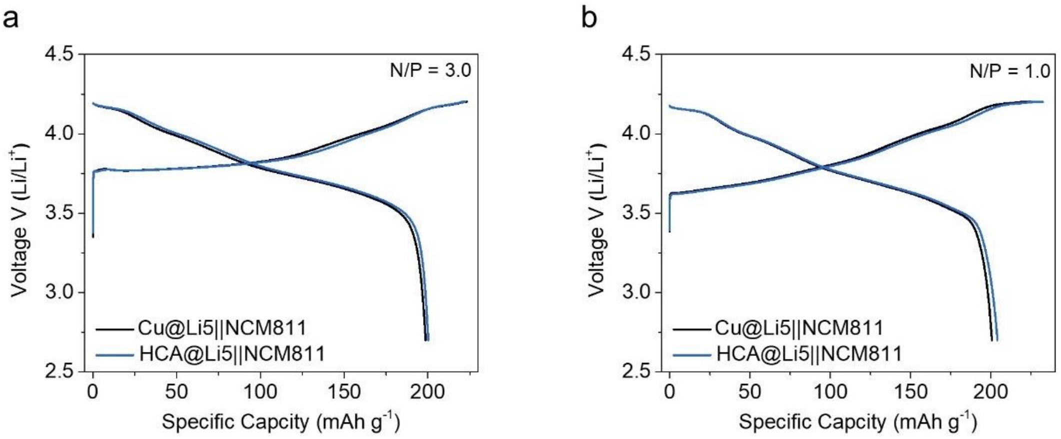

Figure S17. Galvanostatic voltage profiles of Cu-Li5||NCM811 and HCA/C-Li5||NCM811 with N/P ratio of (a) 3.0 and (b) 1.0.

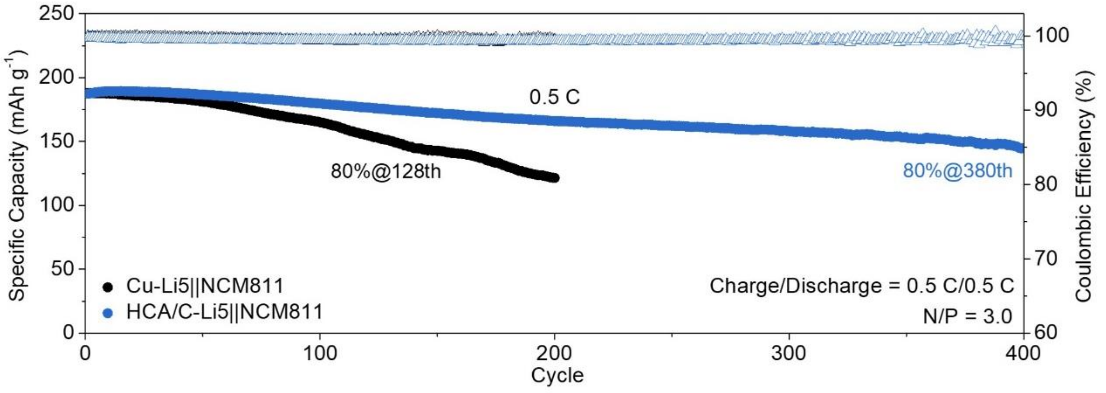

Figure S18. Cycle performance of Cu-Li5||NCM811 and HCA/C-Li5||NCM811 (1 C = 1.7 mA cm^{-2}).

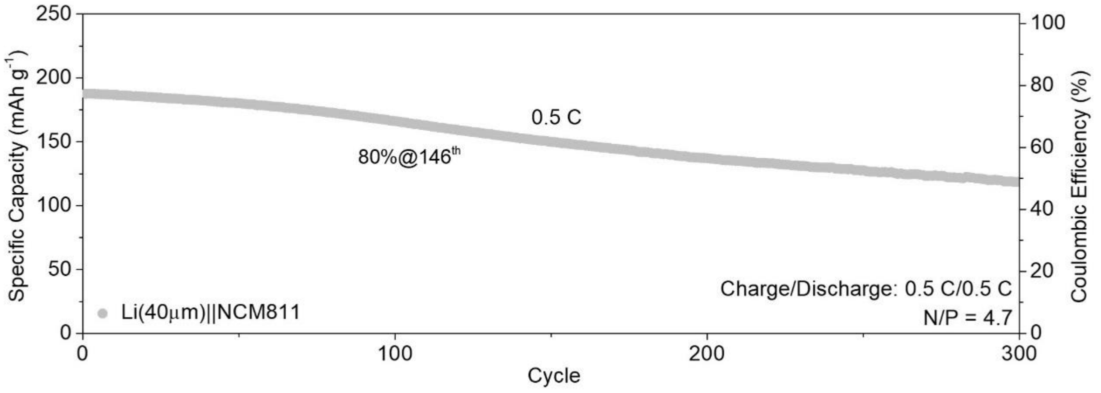

Figure S19. Cycle performance of Li||NCM811 with N/P ratio of 4.7 (1 C = 1.7 mA cm^{-2}).

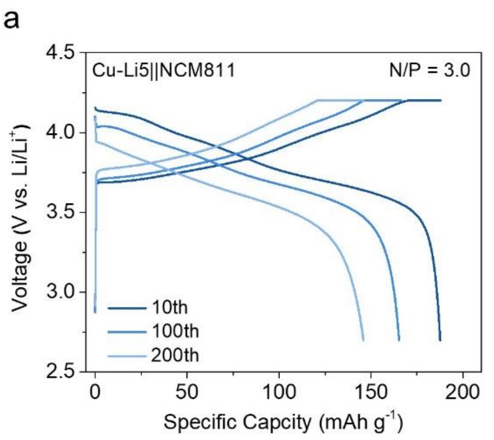

Figure S20. Voltage profiles of selected cycles from (a) Cu-Li5||NCM811 and (b) HCA/C-Li5||NCM811.

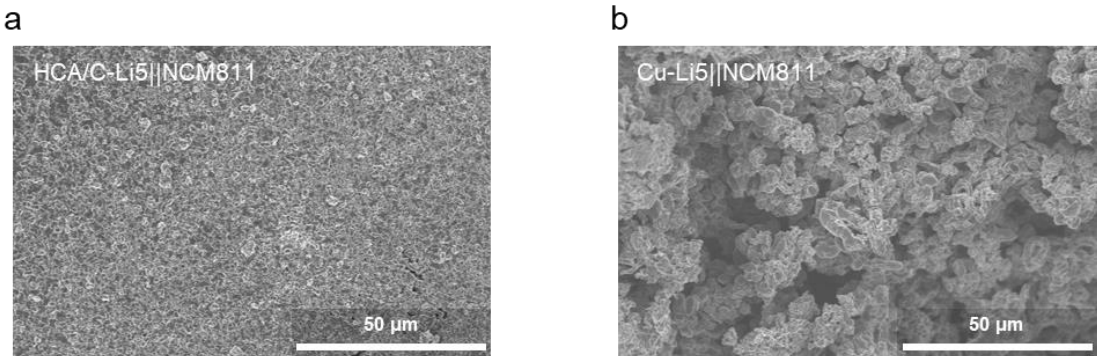

Figure S21. SEM images of (a) HCA/C-Li and (b) Cu-Li after 70 cycled in the full cells.

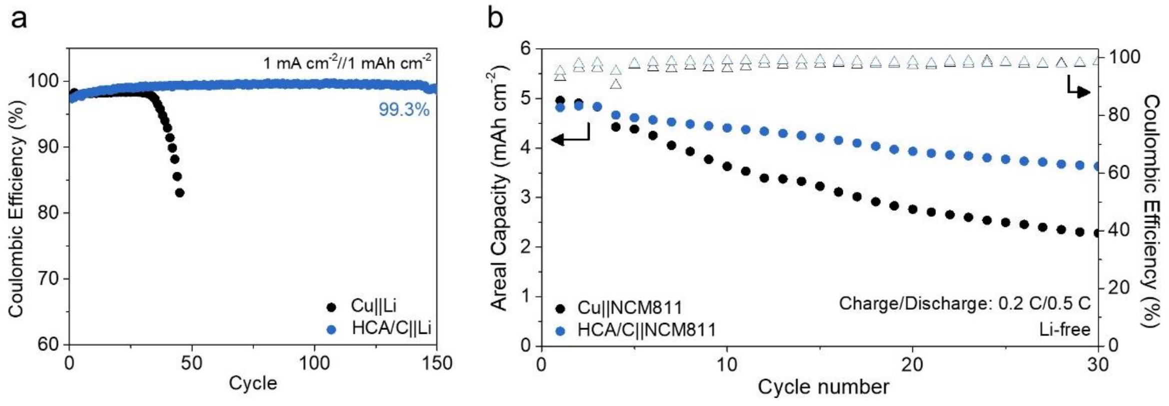

Figure S22. (a) Cycle efficiency for lithium electrodeposition/dissolution on Cu and in HCA/C under the current density of 1 mA cm^{-2} for areal capacity of 1 mAh cm^{-2}. (b) Cycle retention of Cu||NCM811 and HCA/C||NCM811 with high-loaded cathode (1 C = 5.0 mA cm^{-2}).

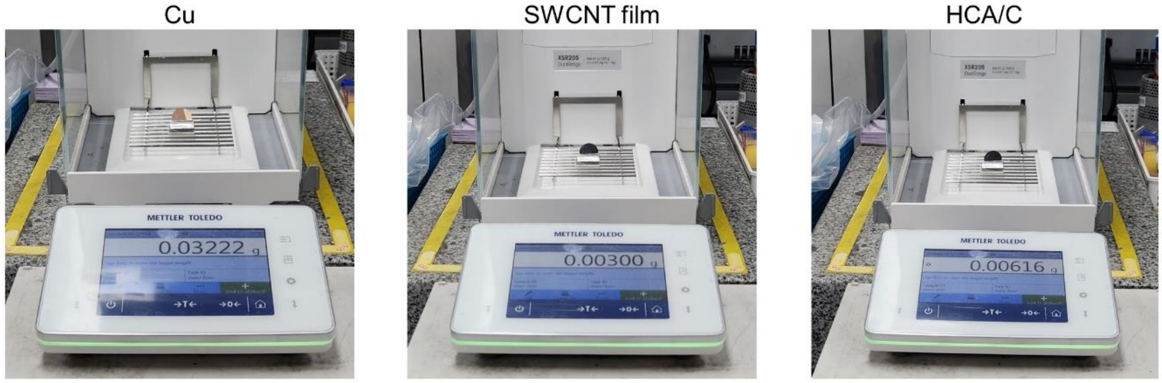

Figure S23. The weight comparison of Cu, SWCNT film, and HCA/C in 16pi disk.

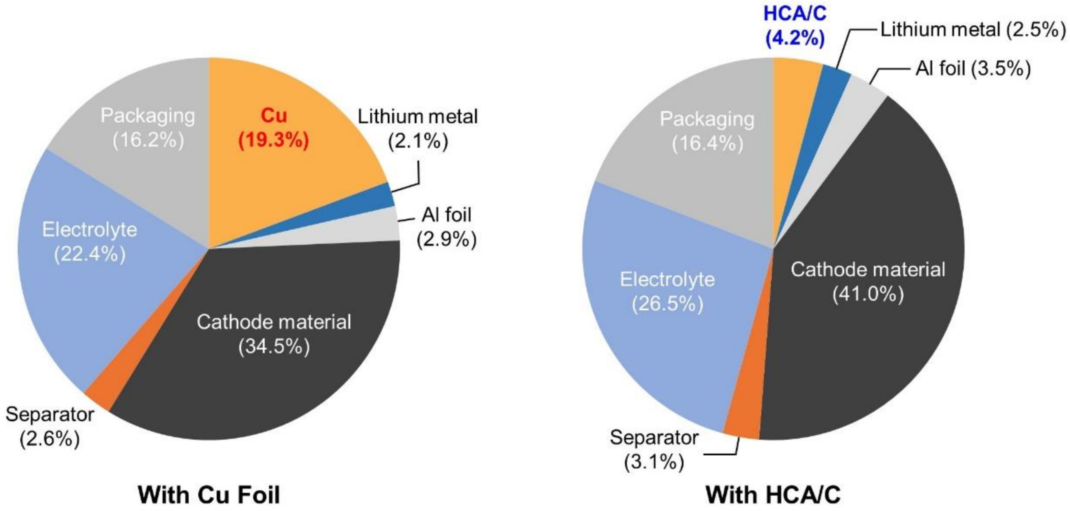

Figure S24. Pie chart comparison of weight contribution for building the stack pouch full cells.

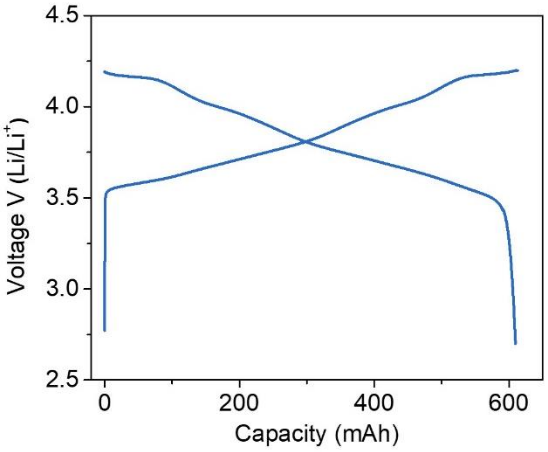

Figure S25. Voltage profile of designed stack pouch cell using HCA/C-Li5||NCM811.

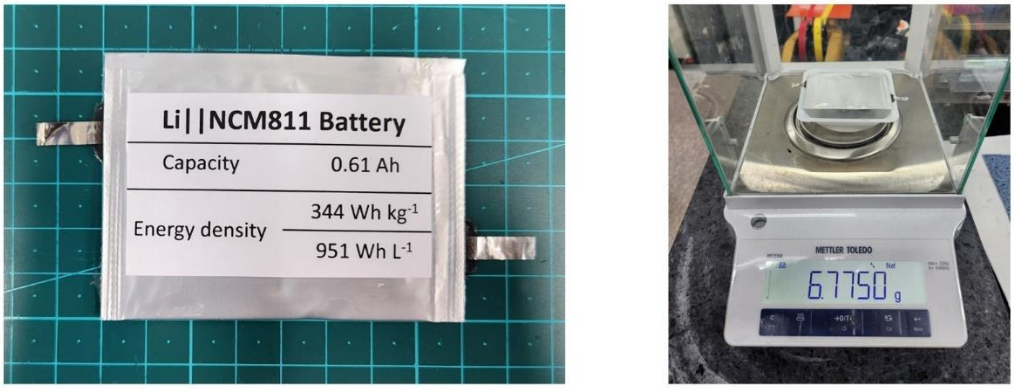

Figure S26. Photograph images of as-assembled stack pouch full cell and total weight measurement of the pouch cell.

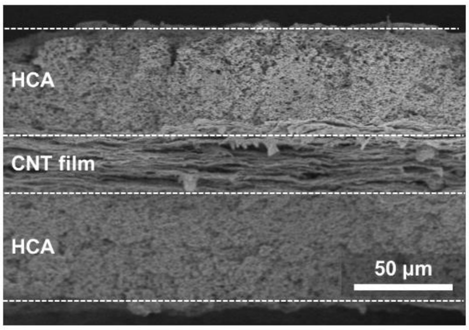

Figure S27. SEM images of HCA/C after double side casting of HCA.

Total Hildebrand parameter, δ_t, can be calculated from the following equation:

$$\delta_t^2 = \delta_d^2 + \delta_p^2 + \delta_h^2$$

Table S1. Hansen solubility parameters and the calculated solubility parameter distance. R_{HSP} values are calculated based on the water (solvent) and other nonsolvents.

	δ_d ($MPa^{1/2}$)	δ_p ($MPa^{1/2}$)	δ_h ($MPa^{1/2}$)	δ_t ($MPa^{1/2}$)	R_{HSP} ($MPa^{1/2}$)
Water	15.6	16	42.3	47.8	
Methanol	15.1	12.3	22.3	29.6	20.4
Ethanol	15.8	8.8	19.4	26.4	24.0
1-Propanol	16	6.8	17.4	24.5	26.6
1-Butanol	16	5.7	15.8	23.1	28.4

Table S2. Comparison of cathode type, cathode mass loading, current density, and N/P ratio of coin-type full cell fabricated in this work and previously reported works.

Cathode type	Cathode mass loading (mg cm^{-2})	Current density (mA cm^{-2})	N/P ratio	Reference
NCM811	23.4	2.5	1.0	This work
NCM811	8.5	0.9	> 5	[1]
LFP	20	1.5	1.33	[2]
LFP	2.54	1.9	7.8	[3]
LCO	4	1.28	N/M	[4]
LFP	5	0.85	5.8	[5]
NCM811	16.6	1.9	1.05	[6]
LFP	16.3	1.25	N/M	[7]
NCM811	12.5	1.25	N/M	[8]

* N/M: Not mentioned.

Table S3. Summarized table of cell components in the stack pouch full cell.

	**Size (W x L, mm)**	**# of sheet**	**Weight (g)**
Anode current collector (Cu foil)	53 x 63	3	1.552

Anode current collector (HCA/C)		3	0.291
deposited Li metal		4	0.169
Cathode current collector (Al foil)	50 x 60	2	0.236
Cathode material		4	2.774
Separator	56 x 66	4	0.208
	**Amounts**		**Weight (g)**
Electrolyte	3.0 g Ah^{-1}	-	1.80
			**Weight (g)**
Package (pouch, tabs)	-		1.299

Table S4. Measured and calculated cell parameters of the stack pouch full cell.

**Parameter**	**Value**	**Unit**
Total weight	6.775	g
Discharge capacity	0.61	Ah
Working voltage	3.82	V
Cell energy	2.33	Wh

Gravimetric energy density	344	Wh kg^{-1}
Volumetric energy density	951	Wh L^{-1}

Table S5. Comparison of the pressure, capacity, and energy density of pouch full cell fabricated in this work and previously reported high-energy-density pouch full cells (Li-free denotes configurations where the anode initially contains no reserve lithium).

N/P ratio	Pressure (kPa)	Capacity (Ah)	Gravimetric energy density (Wh kg^{-1})	Volumetric energy density (Wh L^{-1})	Reference
**1.0**	**20**	**0.61**	**344**	**951**	**This work**
Li-free	250	0.25	325	N/M	[9]
Li-free	2000-4000	0.6	N/M	942	[10]
Li-free	54.4	2.46	320	850	[11]
1.7	500	0.048	321	772	[12]
2.6	69.0	1.0	300	N/M	[13]
1.5	N/M	3.5	340	N/M	[14]
2.96	N/M	0.9	300	N/M	[15]
5.0	N/M	N/M	260	N/M	[16]
6.0	N/M	0.048	59	N/M	[17]

*N/M: Not mentioned.

References

[1] S. Y. Ni, M. T. Zhang, C. Li, R. H. Gao, J. Z. Sheng, X. Wu, G. M. Zhou, *Adv. Mater.* **2023**, 35, 2209028.

[2] C. Y. Wang, C. P. Yang, Y. H. Du, Z. P. Guo, H. Ye, *Adv. Funct. Mater.* **2023**, 33, 2303427.

[3] N. N. Zhang, L. L. Du, J. Y. Zhang, H. T. Xu, X. Zhou, L. Q. Mai, L. Xu, *Adv. Funct. Mater.* **2023**, 33, 2210862.

[4] W. Y. Chen, S. P. Li, C. H. Wang, H. Dou, X. G. Zhang, *Energy Environ Mater* **2023**, 6, e12412.

[5] H. N. Lin, Z. W. Zhang, Y. D. Wang, X. L. Zhang, Z. X. Tie, Z. Jin, *Adv. Funct. Mater.* **2021**, 31, 2102735.

[6] M. H. Ryou, S. H. Kim, S. W. Kim, S. Y. Lee, *Energy Environ. Sci.* **2022**, 15, 2581.

[7] Y. Y. He, L. B. Song, Z. D. Li, X. Y. Yao, Z. Peng, *Nano Energy* **2023**, 118, 109027.

[8] S. W. Li, S. Zhang, C. C. Sun, W. Y. Zhao, T. Zhao, M. Zhang, H. L. Wang, Y. Ma, *Energy Storage Mater.* **2021**, 35, 378.

[9] Z. Yu, H. S. Wang, X. Kong, W. Huang, Y. C. Tsao, D. G. Mackanic, K. C. Wang, X. C. Wang, W. X. Huang, S. Choudhury, Y. Zheng, C. V. Amanchukwu, S. T. Hung, Y. T. Ma, E. G. Lomeli, J. Qin, Y. Cui, Z. N. Bao, *Nat. Energy* **2020**, 5, 526.

[10] Y. G. Lee, S. Fujiki, C. Jung, N. Suzuki, N. Yashiro, R. Omoda, D. S. Ko, T. Shiratsuchi, T. Sugimoto, S. Ryu, J. H. Ku, T. Watanabe, Y. Park, Y. Aihara, D. Im, I. T. Han, *Nat. Energy* **2020**, 5, 299.

[11] Y. Qiao, H. J. Yang, Z. Chang, H. Deng, X. Li, H. S. Zhou, *Nat. Energy* **2021**, 6, 653.

[12] J. H. Kim, J. M. Kim, S. K. Cho, N. Y. Kim, S. Y. Lee, *Nat. Commun.* **2022**, 13, 2541.

[13] C. J. Niu, H. Lee, S. R. Chen, Q. Y. Li, J. Du, W. Xu, J. G. Zhang, M. S. Whittingham, J. Xiao, J. Liu, *Nat. Energy* **2019**, 4, 551.

[14] X. Q. Zhang, T. Li, B. Q. Li, R. Zhang, P. Shi, C. Yan, J. Q. Huang, Q. Zhang, *Angew. Chem. Int. Ed.* **2020**, 59, 3252.

[15] Y. L. Gao, M. Y. Guo, K. Yuan, C. Shen, Z. Y. Ren, K. Zhang, H. Zhao, F. H. Qiao, J. L. Gu, Y. Q. Qi, K. Y. Xie, B. Q. Wei, *Adv. Energy Mater.* **2020**, 10, 1903362.

[16] M. S. Kim, J. H. Ryu, Deepika, Y. R. Lim, I. W. Nah, K. R. Lee, L. A. Archer, W. I. Cho, *Nat. Energy* **2018**, 3, 889.

[17] J. Y. Chen, S. J. Li, X. Qiao, Y. Z. Wang, L. N. Lei, Z. Y. Lyu, J. Zhao, Y. Zhang, R. Q. Liu, Q. H. Liang, Y. W. Ma, *Small* **2022**, 18, 2105999.
